# *Wnt4* is heterogeneously activated in maturing β-cells to control calcium signaling, metabolism and function

**DOI:** 10.1038/s41467-022-33841-5

**Published:** 2022-10-21

**Authors:** Keiichi Katsumoto, Siham Yennek, Chunguang Chen, Luis Fernando Delgadillo Silva, Sofia Traikov, Dror Sever, Ajuna Azad, Jingdong Shan, Seppo Vainio, Nikolay Ninov, Stephan Speier, Anne Grapin-Botton

**Affiliations:** 1 Max Planck Institute of Molecular Cell Biology and Genetics (MPI-CBG), Dresden, Germany; 2 Novo Nordisk Foundation Center for Stem Cell Biology (DanStem), University of Copenhagen, Copenhagen, Denmark; 3 Institute of Physiology, Faculty of Medicine, Technische Universität Dresden, Dresden, Germany; 4 Paul Langerhans Institute Dresden of the Helmholtz Zentrum München at the University Clinic Carl Gustav Carus of Technische Universität Dresden, Helmholtz Zentrum München, Neuherberg, Germany; 5 German Center for Diabetes Research, München-Neuherberg, Germany; 6 Center for Regenerative Therapies (CRTD), TU Dresden, Germany; 7 Faculty of Biochemistry and Molecular Medicine, Biocenter Oulu, University of Oulu, Oulu, Finland

**Keywords:** Genetics, Homeostasis, Endocrinology, Cell signalling

## Abstract

Diabetes is a multifactorial disorder characterized by loss or dysfunction of pancreatic β-cells. β-cells are heterogeneous, exhibiting different glucose sensing, insulin secretion and gene expression. They communicate with other endocrine cell types via paracrine signals and between β-cells via gap junctions. Here, we identify the importance of signaling between β-cells via the extracellular signal WNT4. We show heterogeneity in *Wnt4* expression, most strikingly in the postnatal maturation period, *Wnt4*-positive cells, being more mature while *Wnt4-*negative cells are more proliferative. Knock-out in adult β-cells shows that WNT4 controls the activation of calcium signaling in response to a glucose challenge, as well as metabolic pathways converging to lower ATP/ADP ratios, thereby reducing insulin secretion. These results reveal that paracrine signaling between β-cells is important in addition to gap junctions in controling insulin secretion. Together with previous reports of WNT4 up-regulation in obesity our observations suggest an adaptive insulin response coordinating β-cells.

## Abstract (web summary)

Diabetes is characterized by loss or dysfunction of pancreatic β-cells. Here the authors show that the signalling protein WNT4 is heterogeneously activated by β-cells during their postnatal maturation with WNT4 positive cells being more mature, and that inactivating WNT4 in β-cells after maturation impairs beta-cell function.

## Introduction

The generation and the maintenance of appropriate numbers of functional β-cells is important to prevent diabetes. β-cells regulate glucose homeostasis by releasing insulin in response to high glycemia. They also secrete insulin in response to other cues such as fatty acids, amino acids, hormones or neural signals via neurotransmitters^1^. Despite these common general features, β-cells are morphologically and functionally heterogeneous^2–6^.

Heterogeneity among β-cells has been observed in the early postnatal period during which β-cells progressively mature, aquiring genes associated with adult β-cell function and state^7,8^, including Ucn3^9^, Flattop/Cfap126^6^, Srf^8^, ST8SIA1^10^ and CD9^10^. It has also been reported in adult β-cells^5,11–13^, where a few of the genes expressed in subpopulations of β-cells have been associated to functional outcomes, notably glucokinase levels with different glucose sensing thresholds^11^, C1 with a proliferative state^14^, IGF-IR with aging^15^, or polysialylated-neural cell adhesion molecule (PSA-NCAM) with higher calcium signaling, ATP levels, Glut2 and Gck expression level as well as insulin secretion^16–19^. It is currently unclear whether this heterogeneity represents fixed populations with dedicated functions or transient states, and the representation of different populations change with age and diabetes progression^20^. These heterogeneous cells are nevertheless connected by gap junctions, which coordinate the response of multiple cells^5,12,13,21^. This notably appears to enable the 1–10% of β-cells which show low Pdx1, Nkx6.1, and insulin expression to act as pacemaker cells for calcium signaling^17^. While many endocrine and paracrine signals connecting multiple endocrine cell types in the islet are known, little is known about paracrine signals linking different β-cells^22^.

Because variants in Tcf7l2, a transcription factor downstream of the canonical Wnt pathway predispose to diabetes^23,24^, we investigated the Wnt ligands expressed in β-cells and their neighbors. Wnts are secreted ligands that can act either at a short distance or close contact^25^. The Wnt pathway is known to control several aspects of pancreas development, exocrine cell expansion, as well as β-cell expansion in the perinatal period^26,27^. Here, we identify one Wnt ligand, WNT4, which is expressed heterogeneously in β-cells in the few days after birth and becomes more highly expressed, though still heterogeneous, as the β-cells mature. Using conditional acute inactivation in 5–7-week mice, we show that WNT4 β-cell expression controls their ability to activate calcium signaling in response to a glucose challenge, as well as metabolic pathways converging to lower ATP/ADP ratios, which reduces their acute insulin secretion in response to glucose.

## Results

### WNT4 is heterogeneously expressed in β-cells

**Fig. 1:**
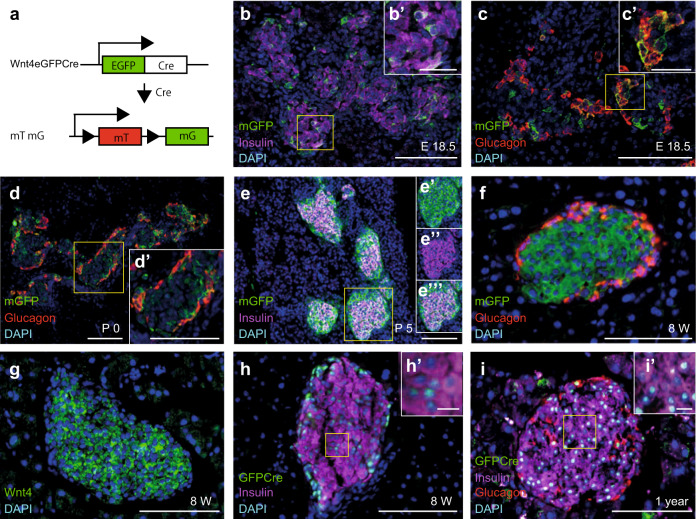
Wnt4 expression in islets. **a** Reporter constructs monitoring *Wnt4* expression (*Wnt4eGFPCre*) and used for lineage tracing (*Wnt4eGFPCre; mTmG*). Expression pattern of Wnt4 revealed by mGFP immunostaining in *Wnt4eGFPCre Tg*+*; mTmG Tg*+ islets at E18.5 (**b, c**), P0 (**d**), P5 (**e**) and 8 weeks (**f**). DAPI marks the nucleus, Insulin β-cells, Glucagon α cells, as color-encoded. Expression of *Wnt4* is detected by WNT4 antibody in 8 weeks WT islet (**g**). Transcription of *Wnt4* (GFPCre) is detected by GFP immunostaining in *Wnt4eGFPCre Tg*+ islets at 8 weeks (**h**) and 1 year (**i**). **b′-d′, h’** and **i′**, High magnification of yellow squares in corresponding panels. **e′–e‴**, split channels of the yellow square in (**e**). Scale bar 100 μm in (**b–i, d′**), 25 μm in (**b′, c′**), and 10 μm in (**h′, i′**). Representative images are from three independent samples.

**Fig. 2:**
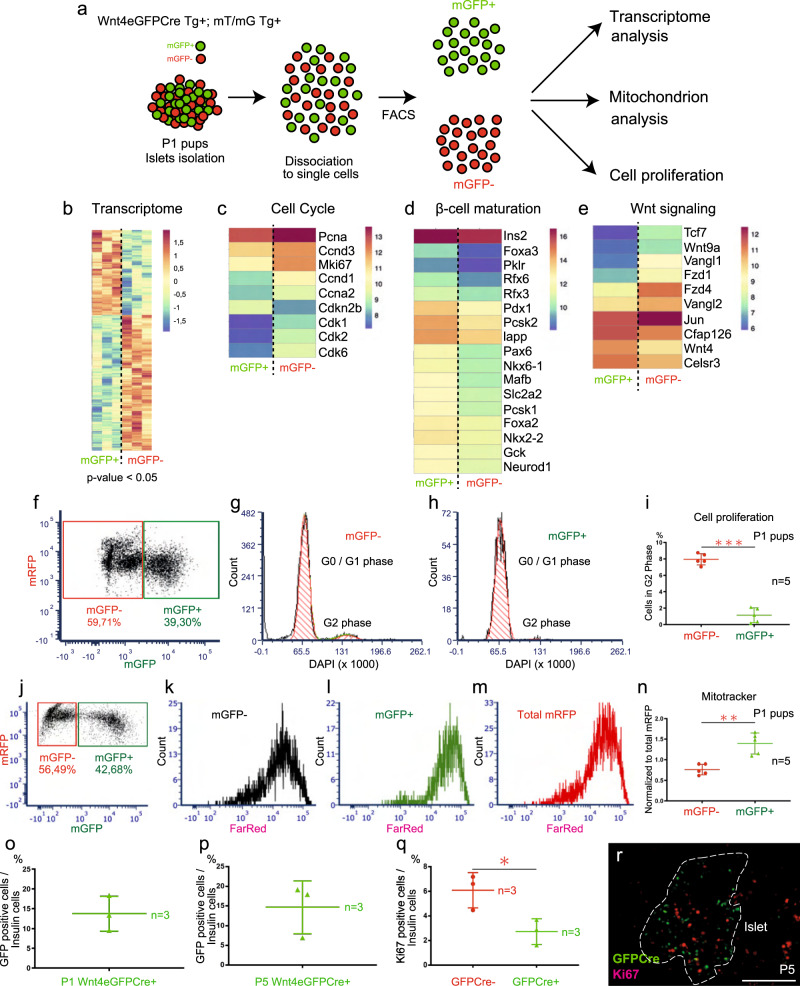
*Wnt4*^low^ cells are more proliferative and *Wnt4*^hi^ cells are more mature. **a** Experimental design relative to **b–n**. **b–e**, Heatmaps showing differentially expressed genes between *Wnt4*^hi^ and *Wnt4*^low^ populations, derived using empirical Bayes statistical analysis (*p* value <0.05). **f–i** Cell proliferation analysis by FACS of sorted mGFP(*Wnt4*)^+^ and mGFP^-^ islet cells. **j–n**, Mitochondrial mass analysis by Mitotracker probe of sorted mGFP^+^ and mGFP^-^ islet cells. **o**, **p** *Wnt4* transcription in P1 and P5 *Wnt4eGFPCre* islets **q, r** Percentage of Ki67 positive cells among GFP^+^ and GFP^−^ cells. **i** The islets from 5 pups were pooled together into 1 sample. In total the islets from 25 pups were used, grouped in samples (*n* = 5). **n** The islets from 5 pups were pooled together into 1 sample. In total the islets from 25 pups were used, grouped in samples (*n* = 5). **o** Three pups samples are analyzed. **p** Three pups samples are analyzed. **q** Three pups samples are analyzed. **r** Scale bar 100 μm. Data in graph of **i**, **n–q** are presented as mean values ± SD. Statistical analyses are two-tailed unpaired student *t*-test. **i** *p* = 8.44e-07, **n** *p* = 0.0012 **q** *p* = 0.0302. *p < 0.05, **p < 0.01, ***p < 0.001. Gene ontology data related to this transcriptome analysis are presented in Supplementary Fig. 1. Supplementary Table 1 complements this figure by providing transcript detection levels in each sample enabling comparisons of the *Wnt4*^hi^ and *Wnt4*^low^ populations. Source data are provided as a Source Data file.

A survey of our and others’ transcriptome analyses revealed that *Wnt4* is transcribed abundantly in the developing pancreas and in islets^28–31^. Using a knock-in tracer allele expressing both eGFP and Cre^32^, we could identify the β-cells that express or have expressed *Wnt4*, using eGFP and Cre-based lineage tracing respectively (Fig. 1a). Expression started in a small subset of β-cells at embryonic day (E) 18.5 (Fig. 1a, b, b′). *Wnt4* was also detected in a subset of α-cells from E12.5 to E18.5 and continued after birth (Fig. 1c, c′, d, d′, f, i). At postnatal day 0 (P0), Cre-driven lineage tracing showed an expansion of Wnt4 (Fig. 1d), reaching around 40% of β-cells by P1 (Fig. 2f, j) and all by P5 (Fig. 1e, e′-e‴). However at P1 and P5, only 15% of β-cells had detectable GFP marking actual transcription suggesting that the transcriptional level varies between cells (Fig. 2o, p, r). WNT4 was not detected at neonatal stages by immunostaining due to a low expression level. By 8 weeks, Cre-tracing confirmed that all β-cells had expressed *Wnt4* and also exhibited detectable WNT4 protein by immunostaining (Fig. 1f, g). However, β-cells exhibited variable transcription, as did α-cells (Fig. 1a, h, h′, i, i′).

### *Wnt4* expression at P1-5 is higher in more mature, less proliferative β-cells

Transcriptome analysis of the *Wnt4*^*hi*^ and *Wnt4*^*low*^ population at P1 (Fig. 2a–e) revealed that gene ontology (GO) categories related to β-cell maturation, function, and cell metabolism were enriched in the *Wnt4*^hi^ population while regulation of cell proliferation and cell adhesion were enriched in the *Wnt4*^*low*^ population (Supplementary Fig. 1a–d and Supplementary Data 1). Proliferation markers such as *Pcna*, *Mki67* and the positive cell cycle regulators *Ccnd1*, *Ccnd3*, *Ccna2*, *Cdk1*, *Cdk2* and *Cdk6* were enriched in the *Wnt4*^*low*^ fraction (Fig. 2c). In agreement, the proportion of cells in the G2 phase of the cell cycle at P1 was higher in the *Wnt4*^*low*^ population (Fig. 2a, f–i). At P5, at the peak of β-cell proliferation^33^, *Wnt4*^*hi*^ cells exhibited lower proportions of the cell cycle marker Ki67 (Fig. 2c, q, r). In contrast, in agreement with the GO analysis, the genes controlling β-cell maturation and function, including transcription factors such as *Pdx1* and *Nkx6.1*, *insulin 2*, *glucokinase* (*Gck*) and the glucose transporter *Slc2a2* were upregulated in the *Wnt4*^hi^ population (Fig. 2d). This population also exhibited a higher mitochondrial abundance (Fig. 2j–n). Taken together, these results show two cell populations, one more proliferative expressing low levels of *Wnt4* and one with higher levels on *Wnt4* exhibiting more functional features of adult β-cells. Because all cells were traced by the Cre recombinase from the *Wnt4-eGFP-Cre* allele, all β-cells must at some point express *Wnt4*, but eGFP intensities at any given time point suggest that *Wnt4* transcription level varies between cells and possibly over time (Figs. 1a, h, h′, i, i′ and 2o, p, r).

Using the transcriptome dataset, we further addressed *Wnt4* expression correlation with canonical and non-canonical Wnt pathway components at P1. It was recently shown that the *flattop/Cfap126* gene is similarly high in a more functional and less proliferative subset of β-cells^6^. In agreement, *Cfap126* was expressed at higher levels in *Wnt4*^*hi*^ cells (Fig. 2e and Supplementary Data 1), in line with the reported enrichment of *Wnt4* in Cfap126^hi^ cells^6^. Though *Cfap126* has been proposed as a readout of the planar-cell polarity pathway (PCP), *Cfap126*^*hi*^*/Wnt4*^*hi*^ cells expressed low levels of *Vgl1/2* and *Jun*. As a functional readout of planar-cell polarity pathway activity, we assessed the Phosphorylation of Myosin Light Chain 2 (pMLC) at P1 by immunostaining. β-cells were positive for pMLC but no correlation was detected between Wnt4 and pMLC at P1 (Supplementary Fig. 2e–h′).

Several transducers of the canonical Wnt pathway, including the *Fzd1* and *Fzd4* receptors as well as transcription factors *Tcf7*, *Tcf7l1* and to a lesser extent *Tcf7l2* were higher in *Wnt4*^*low*^ cells (Fig. 2e and Supplementary Data 1). Since the canonical WNT pathway promotes proliferation in the perinatal period^27^, and since WNT4 inhibits Canonical Wnt signal in β-cell lines^29^ we further assessed the canonical Wnt pathway using immunostaining for the active, dephosphorylated beta-catenin at P1. Though β-cells were positive for active beta-catenin, there was no significant relation between Wnt4 and active beta-catenin at P1 (Supplementary Fig. 2a–d″).

### *Wnt4* conditional inactivation in β-cells results in impaired β-cell function

**Fig. 3:**
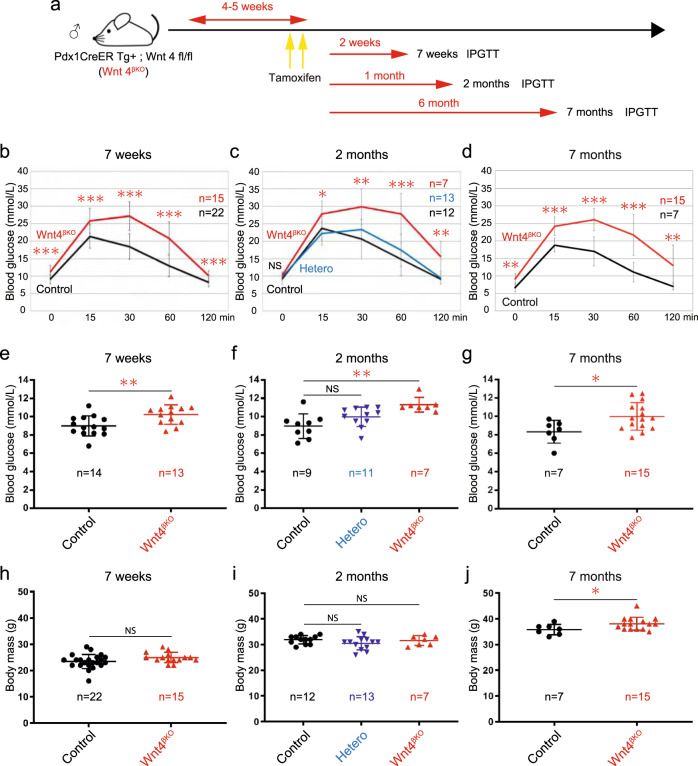
WNT4 in β-cells controls glucose homeostasis. **a** Experimental design of tamoxifen-induced *Wnt4* inactivation in β-cells in male mice and its effect on IPGTT at 7 weeks (**b**), 2 months (**c**) and 7 months (**d**). Basal non-fasted morning blood glucose levels at 7 weeks (**e**), 2 months (**f**), and 7 months (**g**). Body mass of *Wnt4*^βKO^ male mice at 7 weeks (**h**), 2 months (**i**), and 7 months (**j**). Similar measurements in females are presented in Supplementary Figs. 4, 5a–e. Data in graph of **b–j** are presented as mean values ± SD. Statistical analyses are two-tailed unpaired student *t*-test. **b–d**, *p* value from 0 time to 120 min are *p* = 0.0003 (0 time, **b**), *p* = 0.0004 (15 min, **b**), *p* = 5.9e−08 (30 min, **b**), *p* = 2.79e−07 (60 min, **b**), *p* = 0.0001 (120 min, **b**), *p* = 0.1858 (0 time, **c**), *p* = 0.0208 (15 time, **c**), *p* = 0.0021 (30 time, **c**), *p* = 6.38e−05 (60 time, **c**), *p* = 0.0064 (120 time, **c**), *p* = 0.0035 (0 time, **d**), *p* = 0.0001 (15 time, **d**), *p* = 1.58e−05 (30 min, **d**), *p* = 0.0002 (60 min, **d**), *p* = 0.0017 (120 min, **d**). **e** *p* = 0.0062, **f** *p* = 0.0011, **g** *p* = 0.0190, **j** *p* = 0.0490. *p < 0.05, **p < 0.01, ***p < 0.001 and NS not significant. Source data are provided as a Source Data file.

**Fig. 4:**
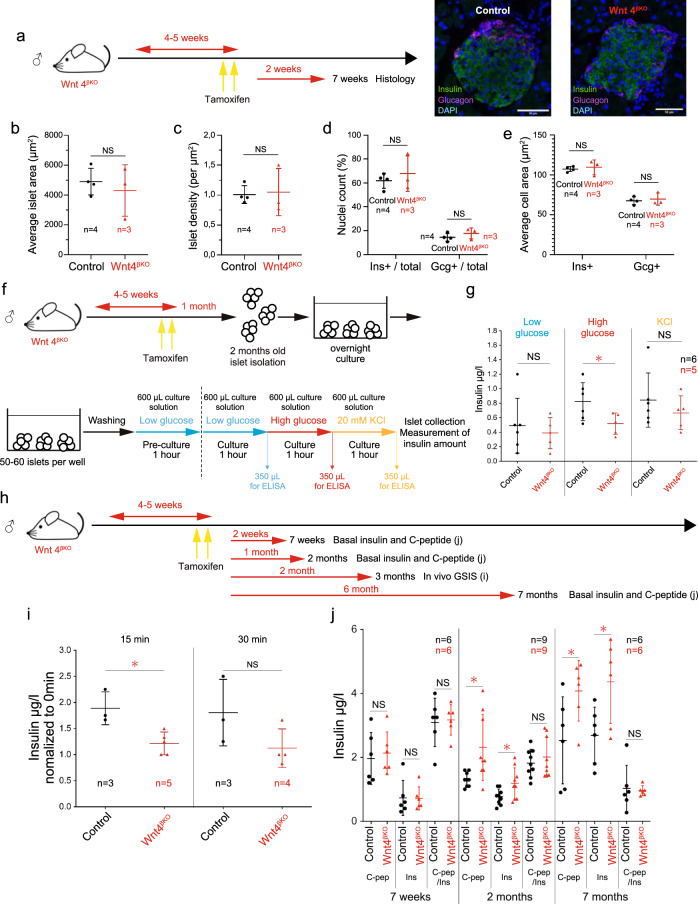
WNT4 controls insulin secretion in β-cells but not their number or size. **a** Experimental design of tamoxifen-induced *Wnt4* inactivation in β-cells in male mice and its effect on islet histology at 7 weeks. Islet area (**b**), and islet density (**c**) % of Insulin^+^ and glucagon^+^ cells (**d**) and average area of insulin^+^ and glucagon^+^ cells (**e**). The average area of islets used for the measurements in (**d**, **e**) is further shown in Supplementary Fig. 5g as the ratios of hormonal cells can be distorted by the size of islets sampled. Experimental design for in vitro GSIS on islets (**f**) and corresponding ELISA measurement of insulin release (**g**). **h** Experimental design for in vivo GSIS shown in (**i**) at 3 months and basal insulin and C-peptide secretion at 7 weeks, 2 months, and 7 months (**j**) upon tamoxifen-induced *Wnt4* inactivation in β-cells in male mice. **i** ELISA for insulin in blood samples from IPGTT of 3 months male mice at 15 and 30 min after glucose injection. **j** ELISA results measuring c-peptide (C-pep) and insulin (Ins) amount in basal blood samples from 7 weeks, 2 months, and 7 months *Wnt4*^*βKO*^ male mice. To check insulin protein maturation efficiency, c-peptide amount is divided by insulin amount at each time point. Data in graph of **b–e**, **g**, **i**, **j** are presented as mean values ± SD. Statistical analyses are two-tailed unpaired student *t*-test. **g** *p* = 0.0464, **i** *p* = 0.0110, **j** *p* = 0.0201 (C-pep, 2 months), *p* = 0.0346 (Ins, 2 months), *p* = 0.0451 (C-pep, 7 months), *p* = 0.0260 (Ins, 7 months). *p < 0.05 and NS not significant. Source data are provided as a Source Data file.

To investigate the function of WNT4, we inactivated it in the β-cells of young adults using *Pdx1-CreER* mice induced with Tamoxifen at 4–5 weeks (Fig. 3a). *Wnt4* transcription was reduced by 40% in *Wnt4* conditional knock-out islets two weeks after triggering inactivation, which is an underestimation as islet preparations contain many non-targeted cell types such as non-β endocrine cells, endothelial cells and contaminating exocrine cells (Supplementary Fig. 4a). Moreover, Wnt4 immunostaining confirmed the extent of the inactivation (Supplementary Fig. 3a–h′). Inactivating *Wnt4* rendered males glucose intolerant as early as 2 weeks after inactivation, with an enhanced effect by the age of 2 months and thereafter (Fig. 3a–d). *Pdx1-CreER;Wnt4 fl/+* heterozygous mice exhibited a normal response to glucose (Fig. 3c). The basal unfasted morning glucose level of tamoxifen-treated *Pdx1-CreER;Wnt4 fl/fl* mice (*Wnt4*^*βKO*^) was also high at 7 weeks, 2 months and 7 months (Fig. 3e–g). Females became glucose intolerant with a delay, starting at 2 months (Supplementary Fig. 4b–f), but there was no difference in basal glucose levels (Supplementary Fig. 4g–i) until 10 months (Supplementary Fig. 4j). There was no significant effect of *Wnt4* inactivation on body mass in the first months in males (Fig. 3h, i) and females (Supplementary Fig. 5a–d) while it was slightly increased after 7 months in males (Fig. 3j) and 10 months in females (Supplementary Fig. 5e). Such physiological defects can be caused either by defective β-cell function or a reduced β-cell mass. We therefore performed a histological assessment of islets at 7 weeks. There was no significant difference between controls and *Wnt4*^βKO^ in terms of islet composition, islet size, and individual endocrine cell size (Fig. 4a–e, Supplementary Fig. 5f, g). Though we have shown that low *Wnt4* expression correlates with a more proliferative state in the perinatal period, deletion in the adult does thus not affect the β-cell mass one may expect if WNT4 controlled proliferation in the adult. This led us to explore a defect in β-cell function.

Glucose-stimulated insulin secretion (GSIS) performed on isolated islets showed reduced insulin release in the *Wnt4*^βKO^ islets in response to high glucose (Fig. 4f, g) and insulin secretion of *Wnt4*^βKO^ mice was also reduced in glucose tolerance tests in vivo (Fig. 4h, i). The unstimulated insulin content in islets was however normal and insulin transcripts were not reduced (Supplementary Fig. 5f, h and Supplementary Data 2). Moreover, insulin and c-peptide were found at normal levels in the blood of *Wnt4*^βKO^ mice at 7 weeks (Fig. 4h, j). This indicates that insulin biosynthesis is not perturbed but its secretion as an acute response to glucose is reduced. In vivo, we observed that the chronically elevated glycemia was accompanied by higher insulinemia suggesting that the islets manage to secrete insulin in a chronic setting at 2 and 7 months. There was no significant difference in the ratio of c-peptide to insulin suggesting a normal insulin uptake (Fig. 4h, j). Although *Wnt4*^βKO^ islets reduced insulin release in GSIS, chronically elevated glycemia was accompanied by higher insulinemia, suggesting impaired insulin sensitivity. To address this possibility, we performed insulin tolerance test and checked serum glucagon and insulin levels (Supplementary Fig. 6a–i). Although *Wnt4*^βKO^ mice showed higher basal unfasted glucose level at 2 months and higher 6 h fasted glucose level at 3 months (Supplementary Fig. 6a–c), there were no significant difference in insulin tolerance at 2 months (Supplementary Fig. 6a, f, g) and 6 h fasted serum insulin and glucagon levels at 3 months (Supplementary Fig. 6a, h, i). These results suggest that insulin sensitivity is not affected in *Wnt4*^βKO^ mice.

### Transcriptional and metabolic analysis shows that WNT4 controls metabolic pathways resulting in lower ATP/ADP ratios in *Wnt4*^βKO^

**Fig. 5:**
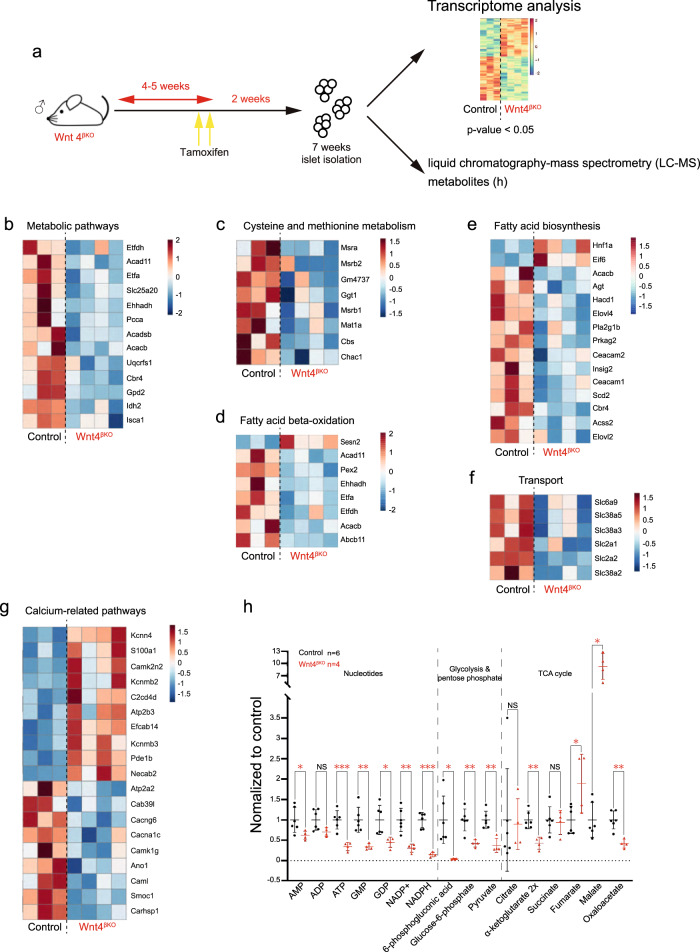
Molecular and metabolic changes in *Wnt4*^*βKO*^ islets. **a** Experimental design for transcriptome analysis and LC–MS analysis of metabolites on different samples. **b–g** Heatmaps showing differentially expressed genes between control and *Wnt4*^*βKO*^ islets as identified in GO analyses of metabolic pathways (**b**), cysteine and methionine metabolism (**c**), fatty acid beta-oxidation (**d**), fatty acid biosynthesis (**e**), transport (**f**) and calcium-related pathways (**g**), derived using empirical Bayes statistical analysis (**a–g** *p* value <0.05). The GO analyses are provided in Supplementary Fig. 7a–d. **h** Metabolite analysis of *Wnt4*^*βKO*^ islets and matched controls by mass spectrometry. Data in graph of **h** are presented as mean values ± SD. Statistical analyses are two-tailed unpaired student *t*-test. **h** p = 0.0486 (AMP), *p* = 0.0006 (ATP), *p* = 0.0024 (GMP), *p* = 0.0184 (GDP), *p* = 0.0017 (NADP+), *p* = 2.26e−05 (NADPH), *p* = 0.0100 (6-Phosphogluconic acid), *p* = 0.0035 (Glucose-6-phosphate), *p* = 0.0012 (Pyruvate), *p* = 0.0016 (α-ketoglutarate 2×), *p* = 0.0278 (Fumarate), *p* = 0.0137 (Malate), p = 0.0012 (Oxalacetate). *p < 0.05, **p < 0.01, ***p < 0.001 and NS not significant. Supplementary Table 2 complements this figure by providing transcript detection levels in each sample enabling comparisons of the *Wnt4*^*βKO*^ islets and matched controls. Source data are provided as a Source Data file.

To determine which genes and pathways are controlled by WNT4 in β-cells, we compared the transcriptome of 7-week islets from mice with *Wnt4*^βKO^ to control littermates (Fig. 5a–g and Supplementary Data 2). Gene ontology and pathway analysis (Supplementary Fig. 7a–d) revealed that *Wnt4*^βKO^ islets showed significant reduction of oxidation-reduction processes and specific metabolic pathways, many of which occurring in the mitochondria, as well as transport mechanisms (Fig. 5b–g and Supplementary Data 2). Among the mitochondrial pathways, cysteine and methionine metabolism, fatty acid beta-oxidation and fatty acid biosynthesis were particularly decreased (Fig. 5c–e, Supplementary Fig. 7a, b and Supplementary Data 2). On the contrary, ribosome-related GO categories and genes, as well as oxidative stress and apoptosis genes were upregulated (Supplementary Fig. 7c, d and Supplementary Data 2). However, no apoptosis was detected by TUNEL assay at this early disease stage.

Our transcription data showed that *Wnt4*^*βKO*^ affects the expression of key metabolic enzymes including mitochondrial isocitrate dehydrogenase 2 (IDH2), and the glucose transporter 2 (SLC2A2/GLUT2) in addition to genes involved in cellular oxidative stress response (Fig. 5b, f). To investigate the metabolic changes associated with *Wnt4*^*βKO*^, we used a targeted approach to measure glycolytic and TCA-cycle metabolites as well as cellular nucleotides using liquid chromatography-mass spectrometry (LC–MS). We observed reduced steady-state levels in Glucose-6-phosphate, pyruvate, oxaloacetate and alpha ketoglutarate in *Wnt4*^*βKO*^ compared to control islets (Fig. 5a, h). In contrast, malate and fumarate levels were increased. This suggests that reduced glucose uptake rate and the transcriptional downregulation of key metabolic enzymes leads to impaired glycolytic and TCA flux affecting cellular energy and precursor availability needed for adult islet function. Indeed, islet ATP/ADP ratio was significantly reduced in mutant islets (Fig. 5a, h). Interestingly, not only Adenosine nucleotides, but most nucleotides including NADP+ and NADPH, key nucleotides needed for cellular redox homeostasis and anabolic reactions, showed reduced steady-state levels (Fig. 5a, h). A reduced flux through the pentose-phosphate pathway (PPP), which is required to nucleotide biosynthesis and cellular redox homeostasis may underlie the observed reduction in cellular nucleotide levels and transcriptional effects on oxidative stress response genes. Strikingly, gluconate an intermediate of the oxidative NADPH-generating branch of the PPP was significantly reduced suggestive of a reduced PPP flux in *Wnt4*^*βKO*^ islets (Fig. 5a, h). Taken together, our data show that the effect of *Wnt4*^*βKO*^ on cell insulin release is accompanied by profound metabolic changes reducing cellular energy and precursor availability, and impaired cellular redox homeostasis.

### Wnt4 additionally controls calcium signaling β-cells

**Fig. 6:**
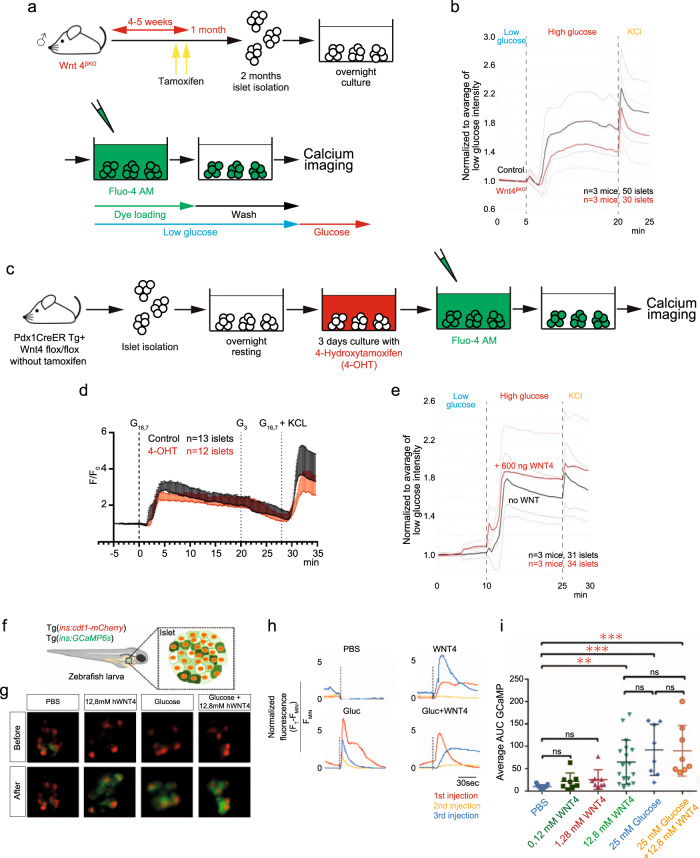
WNT4 promotes influx of calcium in islets of mouse and zebrafish as well as insulin secretion. **a** Experimental design for ex vivo calcium imaging in *Wnt4*^*βKO*^ islets and matched controls. **b** Average calcium fluorescent traces in islets. Area Under Curve (AUC) quantifications are presented in Supplementary Fig. 8a–d. **c** Experimental design for in vitro calcium imaging. **d** Averaged single-cell recordings of intracellular calcium in control and upon *Wnt4* inactivation (4-OHT). Single traces are provided in Supplementary Fig. 8j, k. **e** Calcium imaging in WT islets in the absence or presence of pretreatment with 600 ng WNT4. AUC and peak value quantifications are presented in Supplementary Fig. 8e–h. Red and black broken lines show standard deviations in (**b**, **e**). **f** Cartoon representing a transgenic zebrafish larva expressing the genetically encoded Ca^2+^ indicator GCaMP6s (green) and the nuclear marker cdt1-mCherry (red) under the insulin promoter. Reprinted by permission from [Springer Nature] [Nature Metabolism] (Leader β-cells coordinate Ca2 dynamics across pancreatic islets in vivo, Victoria Salem et al.), [COPYRIGHT] (2019), advance online publication, 14 June 2019 (doi: 10.1038/sj.[NAT. METAB.].) **g** Images from time lapse recording of the islet before and after pericardial injection of 5 nL PBS, 5 nL of 0.5 ng/nL hWNT4 (human), 5 nL of 25 mM glucose and 5 nL of a mixture of 25 mM glucose and 0.5 ng/nL hWNT4. **h** Examples of normalized GCaMP6 traces for each of 3 consecutive injections at 5-min intervals. **i** Quantification of the AUC. Each data point represents the average AUC from three injections in individual larvae. *n* = 13 larvae (PBS), *n* = 8 larvae (0.12 mM WNT4), *n* = 8 larvae (1.28 mM WNT4), *n* = 17 larvae (12.8 mM WNT4), *n* = 8 larvae (25 mM glucose), *n* = 8 larvae (25 mM glucose + 12.8 mM WNT4). *p* < 0.01 (PBS versus 12.8 mM WNT4), *p* < 0.001 (PBS versus 25 mM glucose), *p* < 0.001 (PBS versus 25 mM Glucose + 12.8 mM WNT4). Data are mean ± s.d. (one-way ANOVA with Tukey’s multiple comparison correction, *P* < 0.05, NS not significant) (**i**). Source data are provided as a Source Data file.

In normal β-cells, an increase in ATP upon glucose sensing stimulates the ATP-sensitive potassium channels and leads to a membrane depolarization that activates L-type voltage-gated Ca^2+^ channels, allowing calcium influx from the extracellular space. Calcium then induces insulin exocytosis. Though no change in the ATP-controlled potassium channel transcripts *Kcnj11* and *Abcc9* were detected, two subunits of the L-type calcium channels involved in insulin release (*Cacna1c* and *Cacng6*) were reduced as well as the calcium signaling genes *Cab39l*, *Caml* and *Camk1g* (Fig. 5g and Supplementary Data 2). Calcium is also a signaling intermediate of WNT4 in other tissues. Indeed, the transduction pathway of WNT4 involves Fzd, LRPs, and ROR2 receptors, of which the G-coupled receptors Fzd1, 3, 4, 5, and 6 were abundant in β-cells^34^. WNT4 can activate 3 different pathways depending on cell types: canonical Wnt/β-catenin^35^, planar polarity (PCP)^36^, and calcium signaling via cAMP^37^. In the transcriptome no evidence of decrease of the β-catenin-mediated canonical pathway or of the PCP were detected. However, *Wnt5b*, which was previously shown to be expressed in the *Cfap126*^*lo*^*/Wnt4*^*lo*^ population^6^ was increased upon *Wnt4* loss, suggesting it is repressed by *Wnt4* (Supplementary Data 2). It is possible that *Wnt5b* compensates for the loss of *Wnt4* since both are known PCP activators. The decrease of calcium signaling components may result from a direct effect of WNT4 on its transduction pathway and may affect glucose-induced calcium signaling. We thus further explored whether WNT4 controlled calcium signaling in β-cells. We initially monitored the calcium response elicited by glucose sensing in *Wnt4*^βKO^ islets and controls using Fluo4-AM (Fig. 6a). We found that *Wnt4*-inactivated islets exhibited a globally lower calcium response at high glucose, both in an assay monitoring Fluo4-AM in islets two weeks after in vivo inactivation (Fig. 6a, b and Supplementary Fig. 8a–d) and in single cells 4 days after in vitro inactivation (Fig. 6c, d and Supplementary Fig. 8i–r). Since WNT4 can activate calcium signaling as an immediate response in other cell types, we tested the effect of exposure of WT islets to additional soluble WNT4 on calcium signaling and observed mildly activated calcium signaling, progressively, in the minutes after exposure (Supplementary Fig. 9a–k). We also investigated possible synergies between glucose- and WNT4-induced calcium signaling. Though the calcium signaling levels induced by WNT4 alone were lower than those elicited by glucose alone (compare Fig. 6e and Supplementary Fig. 9a), subsequent exposure to glucose after 15 min exposure to WNT4 accelerated and increased the calcium response to high glucose (Fig. 6e and Supplementary Fig. 8e–h). To test whether this response was relevant in vivo and conserved in the animal kingdom we also injected WNT4 to transgenic zebrafish larvae expressing the genetically encoded Ca^2+^ indicator GCaMP6s (Fig. 6f–i and Supplementary Fig. 9l). As in mouse islets, we found that WNT4 could activate calcium signaling in β-cells on its own, although we couldn’t detect synergies between high glucose and WNT4 in zebrafish larvae (Fig. 6i and Supplementary Fig. 9l). Taken together, WNT4 can activate calcium in β-cells in multiple species within minutes and inactivation shows that it is needed for their calcium response to glucose.

## Discussion

The response of β-cells to elevated glucose levels is regulated by signals from other cells such as neurons, α- and δ-cells, and intestinal L- and K-cells. Several of these signals converge on G-coupled receptors that either increase (GCG from α-cells, GLP-1 from L-cells and α-cells, GIP from K-cells) or decrease (Noradrenalin from neurons, SST from δ-cells) cAMP and eventually intracellular calcium and thereby participate in controlling insulin release^11^. We show here that a signal made by β-cells, WNT4, also activates calcium and contributes to lowering blood glucose. Males were most affected by *Wnt4* inactivation, as often reported in other gene inactivation impairing glucose homeostasis^38^. *Wnt4* has previously been shown to be upregulated in β-cells in obese mice^29^ and under high-fat diet conditions^39^. It is also induced by Exendin-4, mimicking GLP-1 activity^40^. This suggests that WNT4 is a physiological sensor in β-cells that acts to adapt their function to the body metabolic status.

While previous studies in cell lines report disparate effects of WNT4 on insulin transcripts and secretion^6,29,39,40^, we show the effect of WNT4 loss or acute exposure on insulin secretion in mouse islets and the importance of its function in an in vivo context. Studies on β-cell lines have shown that WNT4 does not activate the canonical WNT pathway and rather represses this pathway when it is exogenously activated by WNT3a^40,41^. Moreover, a modest increase in pJNK reported in β-cell lines exposed to WNT4 suggests that it can trigger the planar-cell polarity pathway^6^. However, we have not detected differential activity of the canonical and planar-cell polarity pathway either between WNT4-expressing cells at P1 and those that do not express it or upon its conditional inactivation in β-cells. Activity in the PCP may have been compensated by *Wnt5b* upregulation in the *Wnt4* knock-out. Our work using both loss and acute exposure to WNT4 shows that WNT4 activates calcium signaling in β-cells. Several FZD receptors known to bind and respond to WNT4^34^ are present on β-cells and detected in our transcriptome analysis. Though we did not directly investigate WNT4 secretion in β-cells, this has been reported in multiple cell types, as well as its binding to FZD3 and 6 receptors^34,42,43^. They are GPCRs eliciting calcium influx from the extracellular space^25^ and can synergize with glucose-induced internal store calcium release. The calcium pathway may also have effects at longer time scales. It is notably known to activate calcineurin in β-cells, leading to transcriptional responses^44^. The inactivation of the calcineurin pathway in β-cells also leads to diabetes though the main activity reported is a defect in β-cell proliferation. The differences in phenotype may however be due to the fact that the deletion was performed from the onset of β-cell differentiation rather than in adults and therefore affected the perinatal proliferative period^45^.

In addition to the effect of WNT4 on calcium, our transcriptome and metabolic profiling shows that WNT4 controls several metabolic pathways in β-cells. Previous work highlighted the importance for metabolic mediators linking β-cells metabolic status to glucose-stimulated insulin secretion (GSIS). In particular, several of the pathways we found impaired upon WNT4 inactivation by metabolic profiling of islets are known to control GSIS. This was notably the case for increased cellular ATP/ADP and NADPH/NADP ratios, as well as anaplerotic pyruvate, sustained activity of cytosolic pyruvate citrate, pyruvate isocitrate cycles and pentose-phosphate pathway activities^46^. While WNT4 exerts a direct regulation of calcium via FZD receptors, these metabolic changes were identified in time scales of 1–2 weeks after inactivation and are expected to rely on a transcriptional response of β-cells downstream of WNT4, possibly via calcineurin. Even though exposure to exogenous WNT4 was previously shown to increase the transcriptional regulator *ESSRG* and oxidative phosphorylation in immature β-cells produced from human embryonic stem cells^31^, these pathways were not affected in our loss of function experiments in mice (Supplementary Fig. 7a–d and Supplementary Data 2). This may be either a species difference or, more likely due to the fact that the previous study was performed in the maturation period while ours show a requirement after the cells have matured.

While these recent experiments have shown that WNT4 controls the metabolic maturation of β-cells from human pluripotent stem cells^31^ our experiments showing that WNT4 is turned on in β-cells as they mature provides an in vivo perspective suggesting that in the early postnatal period WNT4 is heterogeneously turned on in β-cells and signals to promote maturation. Moreover they show the important role of WNT4 as a paracrine signal controlling β-cell function and glucose homeostasis in juveniles and adults.

## Methods

### Ethics statement

All experiments with mice conducted in Germany were performed under license 2014-15-2934-01008 and 2019-15-0201-01613 from the Danish Veterinary Office and the license of Tierversuchsvorhabens (TVV 9/2020, “Wnt4 im Pankreas”) from the Governmental IACUC (“Landesdirektion Sachsen”). The experiments with mice conducted in Denmark were approved by the Danish Animal Experiments Inspectorate (Dyreforsøgstilsynet), the Governmental IACUC (“Landesdirektion Sachsen”) and the Max Planck institute Animal Welfare Officer.

All experiments with Zebrafish were carried out in compliance with European Union and German laws (*Tierschutzgesetz*) and with the approval of the TU Dresden and the Landesdirektion Sachsen Ethics Committees (approval no: TVV 45/2018). In this study, all live imaging in vivo, compound and glucose injections, as well as experimental procedures were performed with zebrafish larvae that did not exceed the 5-dpf stage, as stated in the animal protection law (TierSchVersV §14). According to the EU directive 2010/63/EU, the use of these earlier zebrafish stages reduces the number of experimental animals, according to the principles of the 3Rs.

### Mice

All mice were maintained in the animal facilities of either University of Copenhagen or of the Max Planck Institute of Molecular Cell Biology and Genetics (MPI-CBG). Mice were housed under standardized pathogen-free conditions with free access to food and water in a 12/12 h light/dark cycle at an ambient temperature of 20-24 °C and 45-65% humidity. These genetically modified mice lines were used: *Tg(Ifp1-cre/Esr1)*^*35.10Dam*^ ^47^ (*Pdx1CreER*), *Wnt4*^*tm1Svo/tm1Svo*^ ^48^ (*Wnt4 fl/fl*), *Gt(ROSA)26Sor*^*tm4(ACTB−tdTomato,−EGFP)Luo*^ ^49^ (*mTmG*), *Gt(ROSA)26Sor (Rosa26LacZ), Wnt4*^*tm2(EGFP/cre)Svo*^ ^32^ (*Wnt4eGFPCre*). The data were collected on males and females. Specific information relative to gender is indicated for each experiment.

### Conditional inactivation of Wnt4 in β-cells using Tamoxifen administration

Tamoxifen (T5648-1G, Sigma) was dissolved in corn oil (C8267, Sigma) to a final concentration of 50 mg/mL. To inactivate *Wnt4* genes in β-cells, *Wnt4 fl/fl* mice (*Wnt4*^*tm1Svo/tm1Svo*^) were crossed with *Pdx1CreER* (*Tg(Ifp1-cre/Esr1)*^*35.10Dam*^) mice to generate *Pdx1CreER;Wnt4 fl/fl* (*Wnt4*^*βKO*^) and littermate *Wnt4 fl/fl* were used as controls. In some experiments (Fig. 3c, f, i), we also used *Pdx1CreER;Wnt4 fl/+* and could not observe a phenotype, lending support to an absence of effect of the *Pdx1CreER* allele. To trace *Wnt4*-inactivated β-cells, *Rosa26LacZ (Gt(ROSA)26Sor)* mice or *Gt(ROSA)26Sor*^*tm4(ACTB−tdTomato,−EGFP)Luo*^ ^49^ (*mTmG*) mice were crossed with *Pdx1CreER;Wnt4 fl/fl* mice to generate *Pdx1CreER;Wnt4 fl/fl;Rosa26LacZ* (*Wnt4*^*βKO*^*;LacZ*) and *Pdx1CreER;Wnt4 fl/fl;mTmG* (*Wnt4*^*βKO*^*;mTmG*). 4-5week-old *Wnt4*^*βKO*^*;LacZ* and *Wnt4*^*βKO*^*;mTmG* mice were treated with 200 μL Tamoxifen solution twice every other day by gavage. These mice were euthanized between 7 weeks and 12 months for collecting islets and pancreas samples. Cre recombination efficiency for Wnt4 allele was checked by qPCR analysis using 7-week-old *Wnt4*^*βKO*^ islets samples and immunostaining for WNT4.

### Intraperitoneal glucose tolerance test (IPGTT) and basal blood glucose level

Mice were fasted for 6 h and injected with 2 g/kg D-glucose intraperitoneally. Blood drops were collected from the tail at 0, 15, 30, 60, and 120 min after glucose injection to measure blood sugar levels. Glucose levels were assessed by using a glucometer (One Touch select Plus Glucometer #10963194). In separate experiments, blood samples were collected at 0, 15, 30 min after glucose injection for checking serum insulin levels. Basal blood samples were collected in the morning without fasting.

### Insulin tolerance test (ITT)

Mice were fasted for 6 h and injected with 0.5 U/kg insulin (NovoRapid® FlexPen® 100 U/mL, Novo Nordisk A/S) intraperitoneally. Blood drops were collected from the tail at 0, 15, 30, 60, and 120 min after insulin injection to measure blood sugar levels. Glucose levels were assessed by using a glucometer (One Touch select Plus Glucometer #10963194).

### Isolation and culture of murine islets

To isolate adult mouse islets, 5 mL of 0.5 mg/mL Collagenase (C9263 Sigma) in RPMI 1640 (21870076, Gibco^TM^) was injected into the pancreatic duct before its harvesting. The collagenase-injected pancreas was transferred to 1 mL Collagenase solution (0.5 mg/mL) in a 15 mL Falcon tube and incubated for 5 min in a 37 °C water bath and subsequently washed with RPMI 1640, shacked vigorously 10 times and centrifuged 1 min at 1000 rpm. After 3 washing steps, the supernatant was discarded and the pellet resuspended in 3 mL Histopaque®1119 (11191, Sigma) and transferred into a 15 mL falcon tube. The following solutions were each added drop-by drop: 3 mL Histopaque®1083 (10831, Sigma), then 3 mL Histopaque®1077 (10771, Sigma) and finally 3 mL RPMI 1640. The islet preparation was centrifuged 20 min at 2500 rpm without breaking at the end. After centrifuging, a ring of islets formed in the gradient and were picked and transferred to RPMI 1640 in a petri dish. Each of islet was hand-picked under a stereomicroscope and cultured in RPMI 1640 supplemented with 10% fetal bovine serum (FBS) and penicillin–streptomycin at 37 °C with 5% CO_2_.

To isolate neonatal islets, the pancreas from neonatal mice (postnatal day1; P1) was transferred to 500 μL of 0.25 mg/mL Collagenase, RPMI 1640 solution in a 2 mL tube and incubated for 12 min at 37 °C in a water bath. To stop enzyme activity, 1.5 mL 0.5% serum in RPMI 1640 was added after incubation and mixed by pipetting and then centrifuged for 2 min at 220 g at 4 °C. After centrifugation, the supernatant was discarded and washed using 1.5 mL of 0.5% serum in RPMI 1640. After 3 washing steps, they were transferred into RPMI 1640 in a petri dish. Each islet was hand-picked under a stereomicroscope and processed for further experiments. Forty to sixty islets were collected per P1 pup.

### Glucose stimulated insulin secretion (GSIS) assay in isolated mouse islets

Islets were isolated from 2-month-old tamoxifen-gavaged *Wnt4*^*βKO*^ male mice and control littermate males (*Pdx1CreERTg-; Wnt4 fl/fl*). Isolated islets were cultured overnight in RPMI 1640 GlutaMAX^TM^ (61870010, Gibco^TM^) with 10% FBS and Penicillin–Streptomycin at 37 °C with 5% CO_2_.

After overnight incubation, islets were washed and pre-incubated in 3.3 mM glucose (basal glucose), pH 7.4 buffered Krebs-Ringer bicarbonate (KBR)^50^ solution with 10 mmol/l HEPES (KRBH) and with 0.1% BSA (Basal glucose KRBH). KBR solution was saturated with 95% O_2_ for 20 min at 37 °C before starting GSIS assay. Per mouse, 50-60 islets were transferred to 1 well in 4-well plates (176740, Thermo Scientific) with 600 μL basal glucose KRBH and washed 1 time by basal glucose KRBH and then pre-incubated for 1 h at 37 °C. After preincubation, islets were washed again with basal glucose KRBH and followed by 1 h incubation with basal glucose KRBH, 16.7 mM glucose KRBH (high glucose KRBH), and 20 mM KCl in basal glucose KRBH to assess insulin secretion under each condition. Three hundred and fifty microliters solution was collected for analysis for each condition. To check the total insulin content in each well, all islets were collected and sonicated in 100 μL water at the end of the experiment. The 25 μL of sonicate was mixed with 75 μL of Acid ethanol (0.18 M HCl in 96% ethanol) and was incubated in 4 °C for 12 h. Secreted insulin in each condition (basal glucose, high glucose and KCl condition) and total insulin content were quantified using a Mercodia Mouse Insulin ELISA kit following the manufacturer’s protocol (10-1247-01, Mercodia).

### Measurements of Insulin, glucagon, and C-peptide in mouse serum

Blood samples were collected from 7 weeks, 2 months, 3 months, and 7 months tamoxifen-gavaged *Wnt4*^*βKO*^ male mice and control littermate males (*Pdx1CreERTg-; Wnt4 fl/fl*) with or without fasting. Blood samples were collected in 1.5 mL tubes and were centrifuged 15 min at 2500 rpm, 4 °C. After centrifuging, supernatant was collected for ELISA analysis. Insulin content was quantified using a Mercodia Mouse Insulin ELISA kit (10-1247-01, Mercodia) and a Ultra Sensitive Mouse Insulin ELISA Kit (90080, Crystal Chem), and Glucagon was quantified using a Mouse Glucagon ELISA Kit (81518, Crystal Chem), and C-peptide was quantified using ALPCO Mouse C-peptide ELISA kit (80-CPTMS-E01, ALPCO) following the manufacturer’s protocol.

### Calcium signaling measurements

After isolating islets from 2-month-old *Wnt4*^*βKO*^ male mice, 20–30 islets were transferred into 30% Matrigel in RPMI 1640-coated μ-Slide 8 well plate (80826, ibidi) with RPMI 1640 GlutaMAX^TM^, 10% FBS and Penicillin–Streptomycin and cultured at 37 °C with 5% CO_2_ overnight. The assays were performed as described in Kenty et al.^51^. All changed media for calcium imaging were pre-warmed at 37 °C in 5% CO_2_ before media changes. Serum-containing culture media were washed and replaced with 2.5 mmol glucose in KRBH with 0.1% BSA (fasting buffer) for Fluo4, AM (F14201, Invitrogen Thermo Fisher Scientific) loading. Prior to measurements, islets were incubated with 5 μmol Fluo4, AM in fasting buffer at 37 °C with 5% CO_2_ for 1 h. Fluo4 AM-stained islets were washed 5 times in fasting buffer for 15 min. For serial glucose stimulations in calcium imaging, islets were incubated in 2.5 mmol glucose in KRBH (fasting buffer) for 5 min as low glucose condition, then changed to 15 mmol glucose in KRBH (high glucose buffer) for 15 min as high glucose condition and final step was changed to 30 mmol KCl in fasting buffer for 5 min to depolarize the cell membrane.

To evaluate the effect of WNT4 on calcium influx in β-cells, 600 ng of Recombinant Mouse Wnt-4 Protein (rmWNT4, 475-WN, R&D Systems) were added when the 2.5 mmol glucose in KRBH was added, for 15 min and maintained during the 15 mmol glucose in KRBH treatment (Fig. 6e and Supplementary Figs. 8e–h, 9a–k). Isolated wild-type (*Pdx1CreERTg-; Wnt4 fl/fl*) islets were stained by Fluo4, AM as specified above. For serial glucose stimulations in calcium signaling, islets were incubated in 2.5 mmol glucose in KRBH for 5 min twice, then changed to 2.5 mmol glucose in KRBH supplemented with 600 ng of rmWNT4 for 15 min, then changed to 15 mmol glucose in KRBH supplemented with 600 ng of rmWNT4 for 15 min and finally to 30 mmol KCl in fasting buffer for 5 min to depolarize the cell membrane. Control wild-type islets were incubated in low and high glucose without rmWNT4.

A Zeiss LSM 780 inverted microscope (Carl Zeiss) and a Zeiss LSM 880 inverted microscope (Carl Zeiss) were used for time lapse imaging of Calcium influx of murine islets. Confocal imaging was done with a 10× objective, capturing one image every 30 s. On *Wnt4*^*βKO*^ islets Calcium influx imaging, 10 confocal images were taken at 2.5 mmol glucose in KRBH condition (low glucose condition for 5 min), then 30 confocal images were taken at 15 mmol glucose in KRBH condition (high glucose condition for 15 min), and then 10 confocal images were taken at 30 mmol KCL in low glucose condition (KCL condition for 5 min). In experiments stimulating islets with exogenous rmWNT4, 10 confocal images were taken at 2.5 mmol glucose in KRBH condition, then 10 confocal imaging were taken after exchanging the 2.5 mmol glucose in KRBH condition, then 30 confocal images were taken at 600 ng rmWNT4 in 2.5 mmol glucose in KRBH condition, then 30 confocal images were taken at 600 ng rmWNT4 in 15 mmol glucose in KRBH condition and then 10 confocal images were taken 30 mmol KCL in 2.5 mmol glucose in KRBH condition. Calcium influx intensity of series of confocal images were analyzed by FIJI software. All calcium influx intensities were normalized by the average intensity of the first 10 confocal images at 2.5 mmol glucose in KRBH condition.

### In vitro inactivation, calcium imaging, and insulin secretion in perifusion chambers

After overnight culture in standard RPMI 1640 medium supplemented with 1% FBS and 8 mM glucose, islets from *Pdx1CreER;Wnt4 fl/fl* mice were cultured with 4-Hydroxytamoxifen (final concentration: 1 μM, Sigma-Aldrich, cat. no. H6278) or without 4-Hydroxytamoxifen for additional three days before insulin secretion assay or calcium imaging.

For cytosolic calcium imaging, cultured islets were embedded in fibrin gels on coverslips. Briefly, fibrin gels were prepared by mixing 3 μL Hanks’ balanced salt solution (HBSS) with 1 μL human fibrinogen (10 mg/mL in HBSS; Sigma-Aldrich), after which three to five islets, together with 1 μL human thrombin (50 U/mL in HBSS; Sigma-Aldrich) to induce fibrinogen polymerization, were placed individually in the gel. The gel-embedded islets were kept in cultured medium until incubated with Fluo4-AM (3.33 μM, Invitrogen, catalog F14201) for 45 min in 3 mM KRBH buffer (137 mM NaCl, 5.36 mM KCl, 0.34 mM Na_2_HPO_4_, 0.81 mM MgSO_4_, 4.17 mM NaHCO_3_, 1.26 mM CaCl_2_, 0.44 mM KH_2_PO_4_, 10 mM HEPES, 0.1% BSA, 3 mM glucose, pH 7.3) at 37 °C in a cell culture incubator. Calcium imaging was performed in a custom-made temperature-controlled perfusion system using a LSM 780 laser scanning microscope system as previously published^52^. Fluo4-AM fluorescence was excited at 488 nm and emission detected at 500–575 nm. Time series recording spanning 40-50 μm of the islet (Z step: 10 μm, stack of 5-6 confocal images with a size of 512 × 512 pixels) was carried out with a sampling rate of 5 s. Data were analyzed using ImageJ (NIH) from sum intensity projections. The average Fluo4-AM fluorescence intensity of individual islet cells was measured in drawn ROIs using Time Series Analyzer plugin (https://imagej.nih.gov/ij/plugins/time-series.html). Changes in cytosolic Ca^2+^ level were calculated and normalized to the average value in the baseline (F/F_0_) recorded in 3 mM KRBH buffer.

### Immunofluorescence staining

Pancreases were isolated from mice at E18.5, P0, P1, P5, 7,8 weeks, 2, 3, 7, 10 months, and 1 year. These pancreas samples were fixed in 4% paraformaldehyde (PFA) in PBS for 2 h at 4 °C with gentle rotation. After 3 washes in cold PBS, samples were transferred to 15% sucrose in 0.12 M phosphate buffer for 2 h at 4 °C with gentle rotation and then samples were embedded in gelatin (7.5% gelatin diluted in 15% sucrose in 0.12 M phosphate buffer). Gelatin blocks were frozen at −65 °C in isopentane and kept at −80 °C. Frozen sections of 8 μm were made. For permeabilizing the tissue, slides were put into PBST (0.1% Tween 20 in PBS) for 10 min. Incubation with blocking buffer (10% Donkey serum in PBST) for 1 h at room temperature to reduce background. Primary antibodies in blocking buffer were applied to the tissue and incubated overnight at 4 °C. After overnight incubation, tissues were washed 3 times with PBST. The tissues were incubated with secondary antibody (Alexa fluor, see Antibody section) and 4′,6-diamidine-2′-phenylindole dihydrochloride (0.1 μg/mL, DAPI) for 1 h at room temperature, followed by 3 washes with PBST. The tissues were mounted in Fluorescence Mounting Medium (DAKO, S302380-2). All Immunofluorescence images were taken using either a Leica SP8 confocal microscope, a Zeiss LSM 880 Airy upright CZ7 confocal microscope, a Zeiss LSM 880 Airy inverted CZ6 confocal microscope and a Leica DM5500 upright wide-field microscope.

### Antibodies for immunohistochemistry

These antibodies were used in this study for immunohistochemistry; Primary antibodies were goat anti-WNT4 (R&D system, AF475, 1:100) (Fig. 1g), rabbit anti-WNT4 (Bioss Antibodies, BS-6134R, 1:100)(Supplementary Fig. 3), guinea pig anti-Insulin (DAKO, A0564, 1:100), mouse anti-Glucagon (Sigma, G2654, 1:800), chicken anti-GFP (Abcam, ab13970, 1:1000), rabbit anti-Ki67 (Abcam, ab16667, 1:100), mouse anti-active beta-catenin (Millipore, 05-665, 1:100), rabbit anti-pMLC (Cell Signaling Technology, 3674, 1:100). Secondary antibodies were donkey anti-mouse Alexa Fluor^TM^ 568 (Thermo Fisher, A10037, 1:1000), donkey anti-mouse Alexa Fluor® 647 (Jackson ImmunoResearch Europe Ltd, 715-605-150, 1:800), donkey anti-chicken Alexa Fluor® 488 (Jackson ImmunoResearch Europe Ltd, 703-545-155, 1:800), goat anti-chicken Alexa Fluor^TM^ 488 (Thermo Fisher, A-11039, 1:1000), donkey anti-goat Alexa Fluor® 488 (Abcam, ab150129, 1:1000), donkey anti-guinea pig Texas red (Abcam, ab6906, 1:300), donkey anti-guinea pig Biotin (Jackson ImmunoResearch Europe Ltd, 706-065-1480, 1:400), donkey anti-rabbit Biotin (Jackson ImmunoResearch Europe Ltd, 711-065-152, 1:200), Streptavidin Alexa Fluor® 647 (Jackson ImmunoResearch Europe Ltd, 016-600-084, 1:1000). Nuclei were stained with DAPI (Sigma, D9542-1MG, 1:10000).

### TUNEL assay

To detect apoptosis cells on pancreas samples on sections, TUNEL assays were performed with ApopTag Peroxidase in situ apoptosis detection kit (Millipore: S7100) followed by the manufacturer’s protocol.

### Islet mass and composition analysis

Pancreatic islet mass and composition was assessed in 8-μm-thick pancreas cryosections stained for insulin (1:10, guinea pig, Agilent, cat. no. IR002), glucagon (1:500, mouse, Sigma, cat. no. G2654), and DAPI (2.5 μg/L, Sigma-Aldrich). Immunostaining was visualized by Alexa Fluor 488 and Alexa Fluor 546 secondary antibodies (1:200; Life Technologies). Images were acquired in a slide scanner (Axio Scan.Z1; Carl Zeiss) for analysis of islet size and density that includes all islets in the sections. Additionally, 19 to 22 islets from each mouse were imaged using an upright laser scanning microscope (LSM 780 NLO; Carl Zeiss, Jena, Germany) with a water dipping objective (W Plan-Apochromat 203/1.0 DIC M27 75 mm; Carl Zeiss) for analysis of islet composition and cell size (Fig. 4d, e and Supplementary Fig. 5g). Quantification of immunohistochemistry was done manually using ImageJ (NIH).

### Fluorescence activated cell analysis and sorting

#### Cell cycle

The islets from 5 pups were pooled together into 1 sample. In total the islets from 25 pups were used, grouped in samples (*n* = 5). Single-cell dissociated P1 *Wnt4eGFPCre; mTmG* islets were stained with the Vybrant™ DyeCycle™ Violet Stain (Invitrogen, #V35003) for cell cycle analysis, following the manufacturer´s instructions. Cells were incubated at 37 °C for 30 min at a final stain concentration of 1 µM in RPMI 1640 medium supplemented with 2% FBS prior flow cytometry analysis.

#### Mitochondrial activity

The islets from 5 pups were pooled together into 1 sample. In total the islets from 25 pups were used, grouped in samples (*n* = 5). Single-cell dissociated P1 *Wnt4eGFPCre; mTmG* islets were stained with the MitoTracker Deep Red FM (Invitrogen, #M22426) to assay mitochondrial activity, following the manufacturer´s instructions. Cells were incubated at 37 °C for 30 min at a final stain concentration of 25 nM in RPMI 1640 medium supplemented with 2% FBS. After incubation time, cells were washed and resuspended in RPMI 1640 medium supplemented with 2% FBS containing 10 µM of DAPI to exclude dead cells, prior flow cytometry analysis.

#### Flow cytometry analysis

FACS analyses were performed on a BD LSR Fortessa analyzer (BD Biosciences) controlled by BD FACSDiva™ v8.0.1 software. Further analysis and quantifications were performed on the FCS Express 6 Flow Research Edition software v6.01 (De Novo Software). Gating procedures are exemplified in Supplementary Fig. 10.

### RT-qPCR

For total RNA extraction, 7-week-old *Wnt4*^*βKO*^ male mice islets were lysed in RLT buffer containing 1% β-mercaptoethanol and total RNA was extracted by using the RNeasy Plus Micro Kit (QIAGEN: 74034) following the manufacturer’s protocol. For generating cDNA from RNA, 1 μg of total RNA, random-primer and SuperScript III reverse transcriptase (Invitrogen: 18080093) were used following the manufacturer’s protocol. Gene expression levels were checked on StepOnePlus real-time PCR system (Applied Biosystems) using Power SYBR Green PCR Master Mix (Applied Biosystems 4367659). *Wnt4* transcript levels were normalized to the housekeeping gene Hprt. qPCR primers; *Wnt4* fw: CCTGCGACTCCTCGTCTTC; *Wnt4* rv: CTCTGGATCAGGCCTTTGAG, *Hprt* fw: GGCCAGACTTTGTTGGATTTG; *Hprt* rv: TGCGCTCATCTTAGGCTTTGT.

### Microarray analysis

Transcriptome analysis of islets of P1 pups *Wnt4eGFPCre; mT/mG* mice and 7-week-old *Wnt4*^*βKO*^ male mice were performed using the array platform SurePrint G3 Mouse Gene Expression v2 8x60K (Agilent Technologies, # G4852B) following the manufacturer’s protocol. RNA was extracted using the RNeasy Plus Micro Kit (Qiagen, # 74034) and RNA concentration and quality were determined on a 2100 Bioanalyser instrument using the Agilent RNA 6000 Pico kit (Agilent Technologies). *Wnt4*^hi^ is used referring to islet cells expressing high levels of GFP based on lineage tracing, that is having expressed *Wnt4* with onset between E18.5 and P1. The native GFPCre could not be used in these experiments because too low for FACS detection at native levels. The islets from 3–4 pups were pooled together into each individual sample. Three replicates were used. 500 pg of purified RNA was transcribed into cDNA by using the Ovation PicoSL WTA System V2 kit (Nugen, #3312-48) and labeled with the SureTag DNA Labeling kit (Agilent technologies, #5190-3400). For 7-week-old *Wnt4*^*βKO*^ islets, 50 ng of RNA was labeled with the low input Quick Amp Labeling Kit (Agilent Technologies, #5190-2305). Following cDNA (1.5 µg) and cRNA (600 ng) hybridization step using the Gene Expression Hybridization kit (Agilent Technologies, #5188-5242), microarray slides were washed and scanned on a SureScan microarray scanner (Agilent Technologies).

The extracted raw data files were analyzed using the R-BioConductor package limma. The background intensities were subtracted out using the *normexp* function with the default offset value of 50, and then normalized between arrays using the quantile normalization method. PCA, clustering and differential expression analysis were performed using limma package itself.

### Gene ontology (GO) analysis

Gene ontology (GO) analysis was performed to study the roles of the differentially expressed mRNAs. To reveal the significantly up- or downregulated pathways of gene expression profile from P1 *Wnt4eGFPCre;mTmG* islets and tamoxifen-treated 7 weeks *Wnt4*^*βKO*^ islets, the David online tool (https://david.ncifcrf.gov) was used. KEGG pathway analysis were used at the functional level. DAVID analysis adopts a modified Fisher’s Exact test (EASE score) to calculate the enrichment in gene annotation. *p* < 0.05 was considered a statistically significant difference.

### Mass spectrometry

The islets of 7-week-old *Wnt4*^*βKO*^ were collected in 100 μL 70% Ethanol with 100 nM chloropropamide as internal standard and were homogenized for 15 min at 4 °C and 300 × *g* in a TissueLyser II (Qiagen) after adding 1/3 volume of 0.5 mm zirconium beads. Next, 10 μL of homogenate solution was isolated for protein quantification by the BCA Protein Quantification Kit (Pierce BCA Protein Assay Kit, Thermo Scientific). The resulting mixture was centrifuged at 13,000 × *g* for 30 min and the supernatant transferred to a new tube. The supernatant was split in two equal parts for nucleotide estimation and carboxylic acid determination. For nucleotide estimation equal volume of acetonitrile was added and applied to LC–MS system.

For the determination of carboxylic acids the following derivatization procedure was performed: the supernatant was speed vacuum dried and resuspended in 15 μL of dH_2_O. Twenty five microliters 1 M EDC and 50 μL 10 mM 4-BNMA were added and incubated for 45 min in 60 °C and quenched with 100 μL of 50 mM acetate and speed vacuum dried. The pellet was incubated for 30 min in 50 μL CAN:H_2_O (50:50 v/v). LC-MSMS analysis were performed.

LC–MS/MS analysis was performed on a high performance liquid chromatography (HPLC) system (*1200 Agilent*) coupled online to G2-S QTof (*Waters*). For normal phase chromatography Bridge Amide 3.5 μL (2.1 × 100 mm) column from Waters and for reverse phase chromatography CORTECS C18, 2.7 (2.1 × 100 mm) column from Waters were used. The following gradient program for the reverse phase was used: Eluent B: from 50 to 100% within 12 min; 100% from 12 to 17 min; equilibration of 50% from 17 to 25 min. The flow rate was set at 0.3 mL/min.

For the normal phase the mobile phase composed of eluent A and eluent B (40% acetonitrile, 0.1 mM ammonium acetate, and 0.01% NH_4_OH) was applied with the following gradient program: eluent B, from 0% to 100% within 18 min; 100% from 18 to 21 min; 0% from 21 to 26 min. The flow rate was set at 0.3 mL/min. The spray voltage was set at 3.0 kV and the source temperature was set at 120 °C. Nitrogen was used as both cone gas (50 L/h) and desolvation gas (800 L/h), and argon as the collision gas. MS^E^ mode was used with Bridge Amide 3.5 μL (2.1 × 100 mm) column in ESI-negative ionization polarity for the detection of the nucleosides. Mass chromatograms and mass spectral data were acquired and processed by MassLynx software (Waters).

### Zebrafish husbandry

Zebrafish were raised in standard conditions at 28 °C. Established transgenic lines used in this study were *Tg(ins:gCaMP6s;cryaa:mCherry)*^53^, *Tg(ins:cdt1-mCherry;cryaa:GFP)*^54^ to allow for a clear separation of the spectra and simultaneous signal recordings from the GCaMP and mCherry channels.

### Zebrafish live imaging

Embryos were treated with 0.003% (200 µM) 1-phenyl-2-thiourea to inhibit pigmentation from 24 h post fertilization onwards. At 4.5 days post fertilization (dpf), the larvae were anaesthetized using 0.4 g l^–1^ Tricaine and mounted in glass-bottom microwell dishes (MatTek Corporation) using 1% low-melting agarose containing 0.4 g l^–1^ Tricaine. Once the agarose was solidified, the dishes were filled with embryonic fish water containing 0.4 g l^–1^ Tricaine. Live imaging was performed on an inverted laser scanning confocal system, ZEISS LSM 780, using a water C-Apochromat 40×/NA 1.2 correction lens. In the *Tg(ins:GCaMP6s);Tg(ins:cdt1-mCherry)* double-transgenic animals, the GCaMP6 and mCherry signals were acquired simultaneously using the 488 nm and 561 nm laser lines. The videos were recorded focusing on a single plane, recording a frame every 0.5 s with an *XY* resolution of 0.12 µm per pixel (512 × 512 pixels). Laser power was maintained as low as possible (<1.5%) to minimize phototoxicity.

### Pericardial injection in zebrafish

Injections were performed as previousy described^19^ using pulled glass pipettes with a 5-nl tip volume calibrated microscopically (3.5-inch Drummond no. 3-000-203-G/X, Sutter pipette puller P-1000). A pneumatic pico-pump (FemtoJet, Eppendorf), injecting pressure 500 hPa and compensation pressure 0 hPa, was used to deliver a 1-s injection into the pericardial cavity in the agarose-mounted larva, assisted by a micromanipulator (InjectMan N2, Eppendorf). Doses were 5 nL PBS, 5 nL of 0.5 ng/nL Recombinant Human Wnt-4 protein (rhWNT4, R&D system, 6076-WN), 5 nL of 25 mM glucose and 5 nL of 25 mM glucose together with 0.5 ng/nL rhWNT4. Injection of vehicle (1× PBS).

### Quantification of GCaMP6 fluorescence intensity in zebrafish images

For the quantification of changes in GCaMP6 fluorescence the cumulative response of all imaged β-cells to the injection was quantified. To this end, the area under the curve (AUC) based on the normalized fluorescence intensity covering 100 frames after the time of injection was calculated. For the normalization of the fluorescence intensity we subtracted only the background from the imaging using the following formula:

$$({F}_{{{{{\rm{T}}}}}}-{F}_{{{{{{\rm{min}}}}}}})/{F}_{{{{{{\rm{min}}}}}}}$$

where *F*_T_ is the integrated fluorescence intensity at a given time and *F*_min_ is the minimum value recorded during the live imaging session.

The larvae were injected with three separate pulses spaced by 5 min each. For each injection, the GCaMP AUC was calculated covering 100 frames after injection (50 s). Then, the average AUC was calculated from the 3 pulses.

### Spatial drift correction of zebrafish images

The red channel (cdt1-mCherry) signal from the β-cell nuclei was used to correct for spatial drift in the green GCaMP6s channel. We employed the Fiji plugin ‘Descriptor-based series registration (2d/3d + t)’ (https://imagej.net/Descriptor-based_registration (2d/3d))^55^, applying the model ‘Rigid (2d)’, with ‘3-dimensional quadratic fit’. A σ of 13 and threshold of 0.03 were applied to the detection of nuclear signal, with a minimum number of three neighbors, redundancy of 1 and a random sample consensus (Ransac) error of 5. Matching across time series was achieved using global optimization.

### Statistical analysis

The Statistical analysis were performed with GraphPad Prism 6 v9.4.0 and Microsoft Excel v16.65 software. The data were presented as mean ± SD values. To determine statistically significant differences (*p* < 0,05), two-tailed unpaired student’s *t*-test was applied for statistical analysis except for WNT4-treated mouse islets (Supplementary Fig. 9a–k) and Zebrafish experiments (see figure legends).

### Reporting summary

Further information on research design is available in the Nature Research Reporting Summary linked to this article.

## Supplementary information

Supplementary Information

Description of Additional Supplementary Files

Supplementary Data 1

Supplementary Data 2

Reporting Summary

Supplementary Table 1

Supplementary Table 2

## Data Availability

Transcriptome data are available at Geo under accession numbers: GSE210237 (P1 GFP+ vs GFP− samples) and GSE210267 (conditional inducible knock-out of Wnt4 in β-cells). Mass spectrometry data are available at MetaboLights under accession number: MTBLS6012. Source data are provided with this paper.

## Source data

Source data
