# Supplementary material for: Wnt4 is heterogeneously activated in maturing β-cells to control calcium signaling, metabolism and function
Source: Nat Commun. 2022 Oct 21;13:6255. doi: 10.1038/s41467-022-33841-5 (PMC9587236; doi:10.1038/s41467-022-33841-5)
Supplement: Supplementary file 1 — Supplementary Information [file 41467_2022_33841_MOESM1_ESM.pdf]

Title: *Wnt4* is heterogeneously activated in maturing β-cells to control calcium signaling, metabolism and function

Keiichi Katsumoto[1,2]*, Siham Yennek[2#], Chunguang Chen[3,4,5#], Luis Fernando Delgadillo Silva[6], Sofia Traikov[1], Dror Sever[2], Ajuna Azad[2], Jingdong Shan[7], Seppo Vainio[7], Nikolay Ninov[4,6], Stephan Speier[3,4,5], Anne Grapin-Botton[1,2,4]*

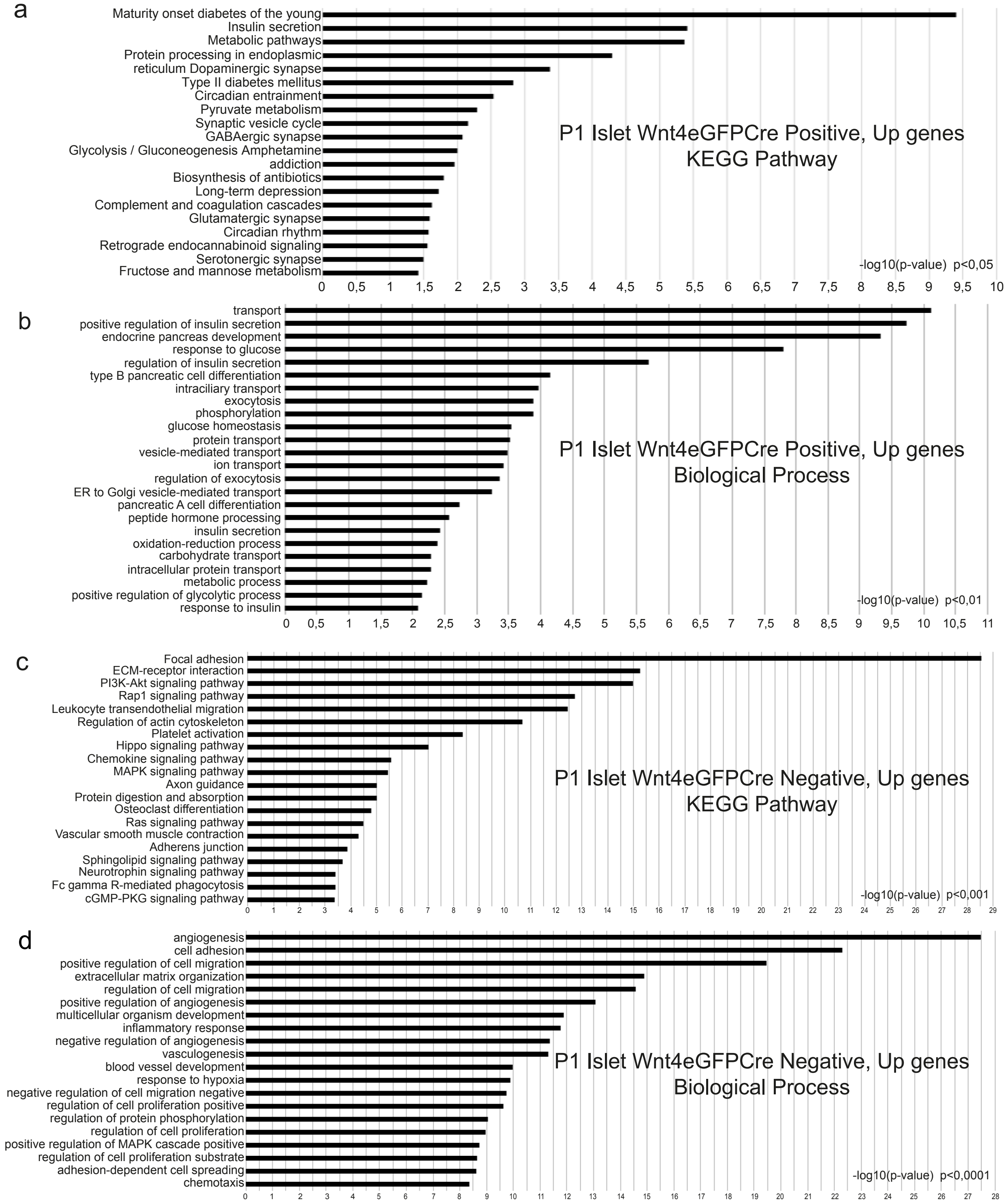

Supplementary Fig. 1: Gene ontology analysis of P1 *Wnt4eGFPCre; mT/mG* islets.
a-d, Gene ontology analysis results of P1 *Wnt4eGFPCre; mT/mG* islets based on DAVID Bioinformatics Resources 6.8. Results with the modified Fisher exact p-value (EASE score) < 0.05 were considered to be significantly enriched. Selected significant enriched terms of gene ontology. Enriched terms of KEGG Pathway (**a**) and Biological Process (**b**) from up-genes of *Wnt4eGFPCre Tg +; mTmG Tg+*. Enriched terms of KEGG Pathway (**c**) and Biological Process (**d**) from up-genes of *Wnt4eGFPCre Tg-; mTmG Tg+*. Source data are provided as a Source Data file.

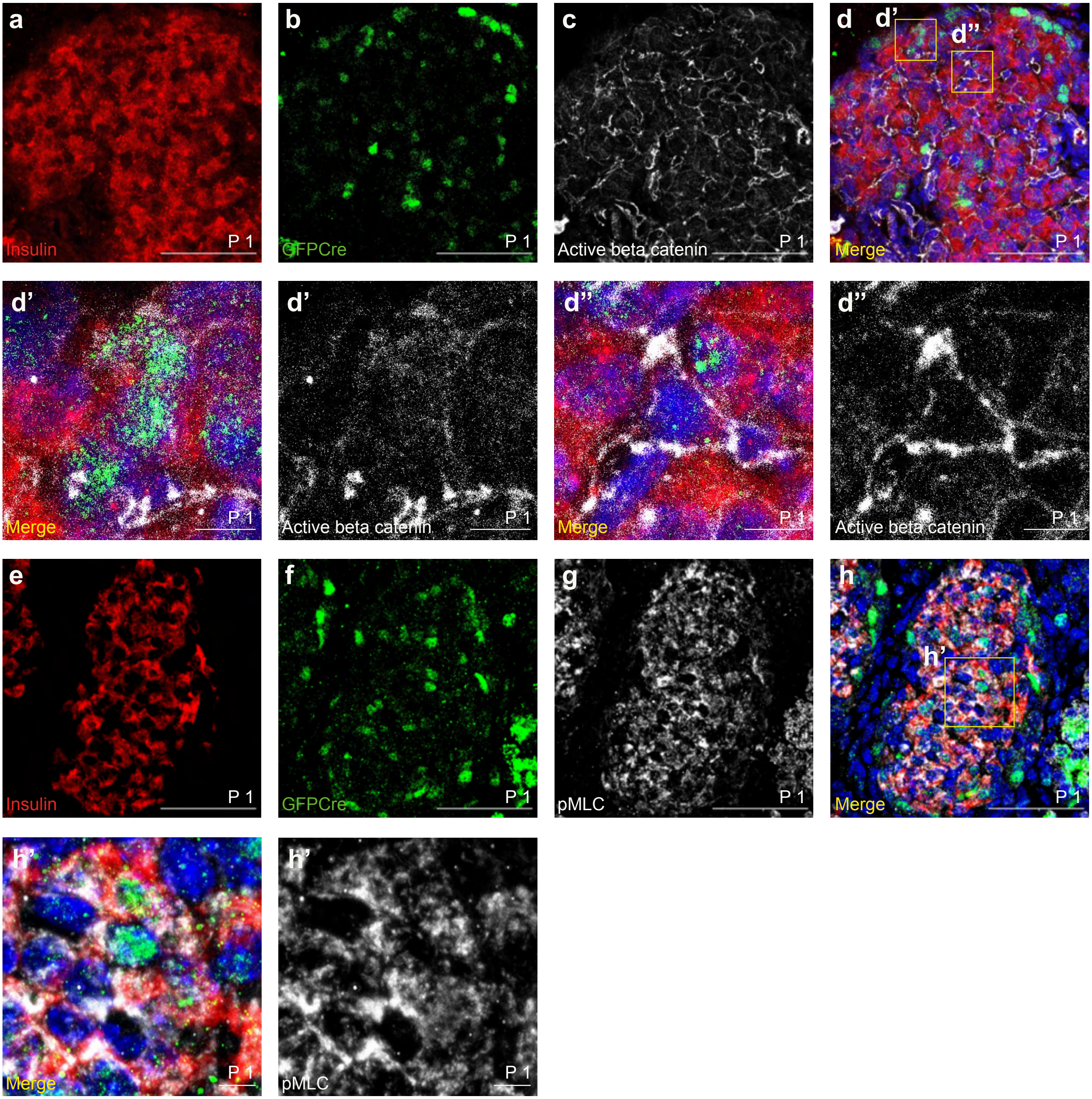

Supplementary Fig. 2: Canonical and non-canonical Wnt signal in P1 islets.
a-d, Expression pattern of insulin (**a**), GFPCre (**b**), active beta-catenin (**c**) and merge image including DAPI staining (**d**) in P1 *Wnt4eGFPCre Tg+* islets. **e-h**, Expression pattern of insulin (**e**), GFPCre (**f**), pMLC (**g**) and merge image including DAPI staining (**h**) in P1 *Wnt4eGFPCre Tg+* islets. **d' d" h',** High magnification of yellow squares in corresponding panels. Scale bar 50 µm in (**a-d, e-h**), 5 µm in (**d', d", h'**). Representative images are from three independent samples.

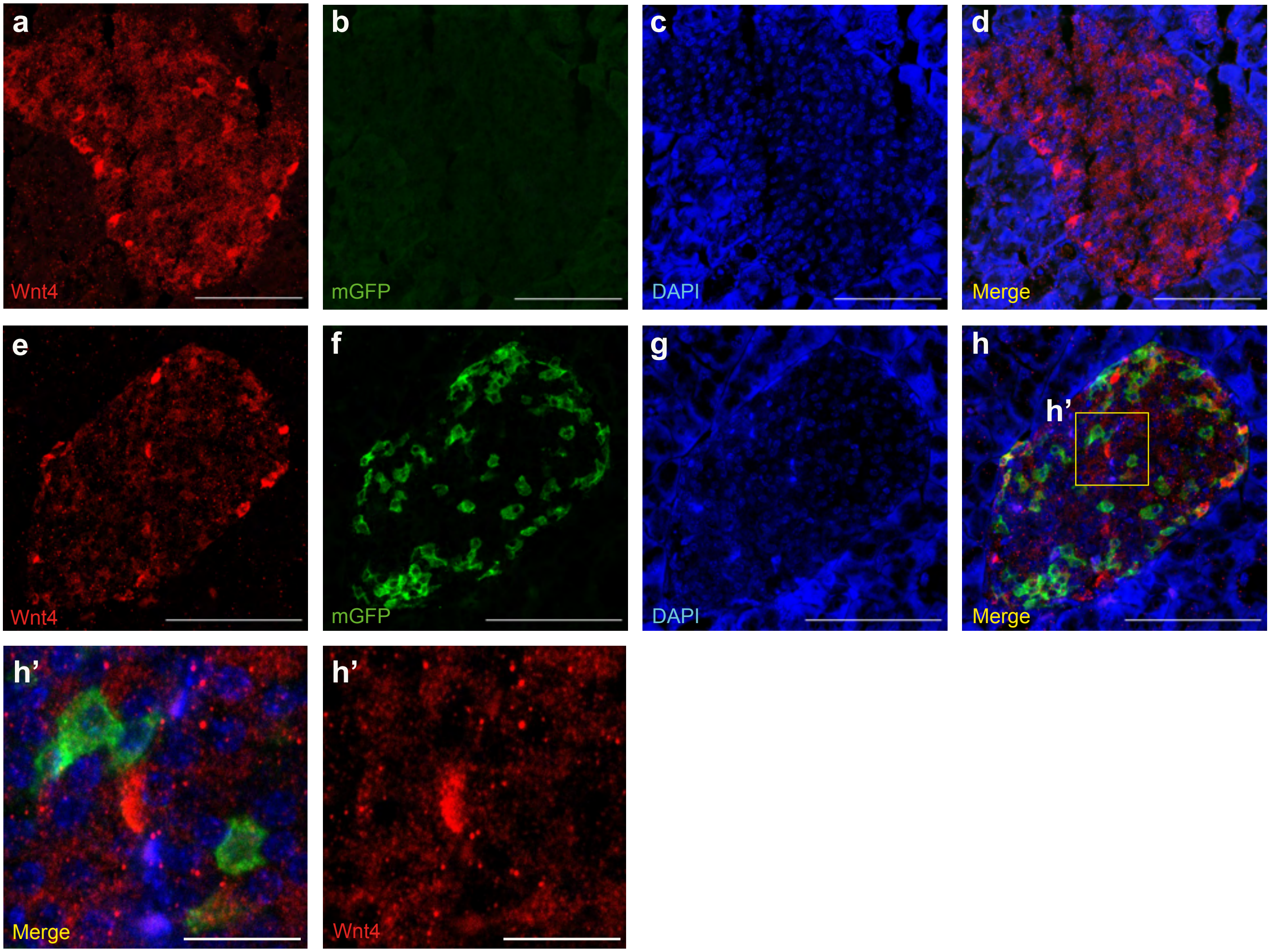

Supplementary Fig. 3: Expression pattern of WNT4 in *Wnt4^{βKO}* islets.
a-d, Expression pattern of Wnt4 (**a**), mGFP (**b**), DAPI staining (**c**) and merge image (**d**) in control 3 months *Wnt4eGFPCre Tg-; mTmG Tg+* islet. **e-h**, Expression pattern of Wnt4 (**e**), mGFP (**f**), DAPI staining (**g**) and merge image (**h**) in mutant 3 months *Wnt4eGFPCre Tg+; mTmG Tg+* islet. **h'**, High magnification of yellow squares in corresponding panels. Scale bar 100 µm in (**a-h**), 20 µm in (**h'**). Representative images are from three independent samples.

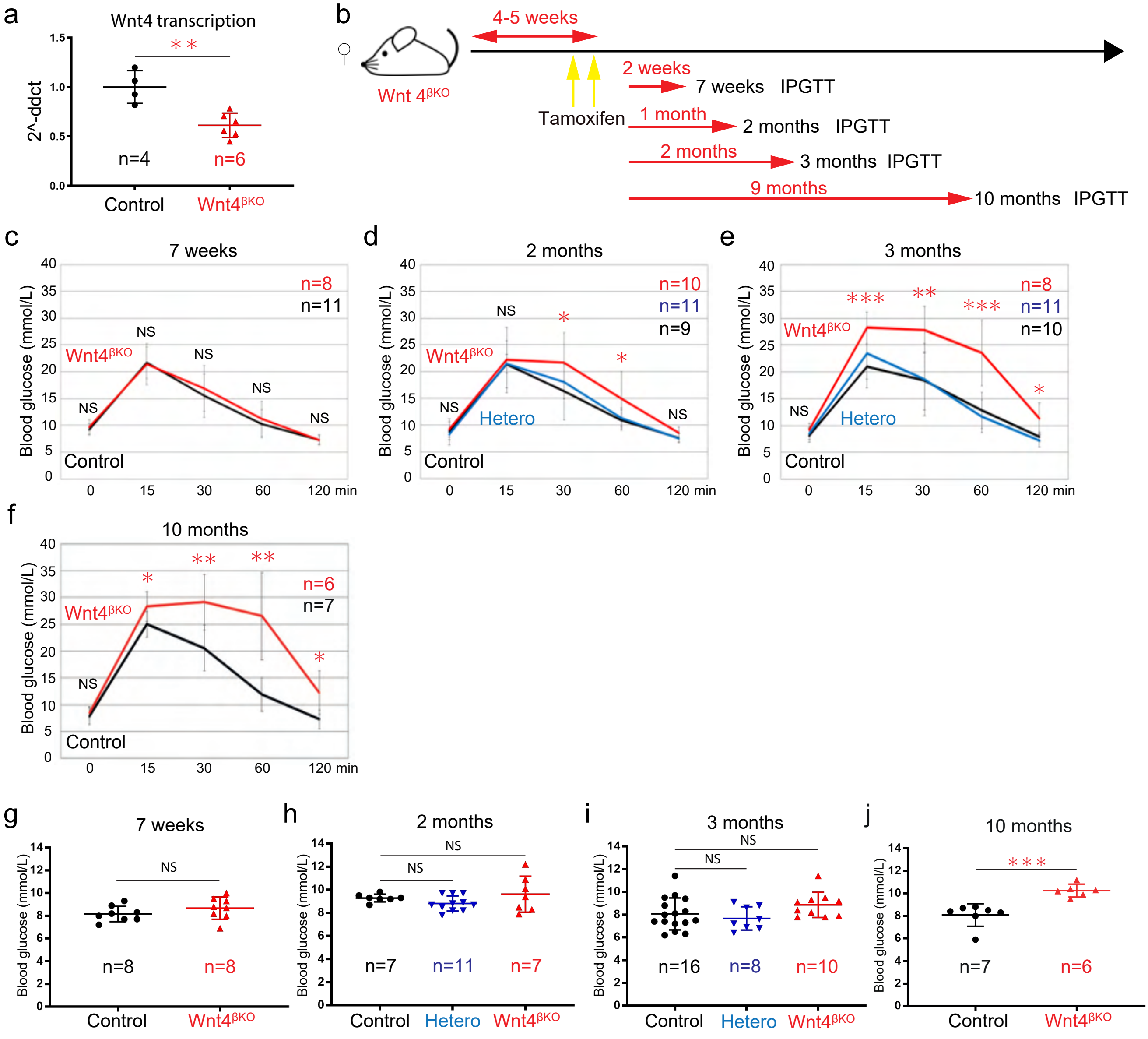

Supplementary Fig. 4: Glucose tolerance and basal glucose in *Wnt4$^{\beta KO}$* females.
a, *Wnt4* qPCR results of 7 weeks *Wnt4$^{\beta KO}$* islets. Control; 4 mice, *Wnt4$^{\beta KO}$*; 6 mice. **b**, Illustration of experimental design for tamoxifen-induced inactivation of *Wnt4* in β-cells in female mice for intraperitoneal glucose tolerance test (IPGTT). **c-f,** Results of IPGTT of *Wnt4$^{\beta KO}$* female mice at 7 weeks (**c**), 2 months (**d**), 3 months (**e**) and 10 months (**f**). **g-j**, Basal blood glucose levels of *Wnt4$^{\beta KO}$* female mice at 7 weeks (**g**), 2 months (**h**), 3 months (**i**) and 10 months (**j**). Data in graph of **a, c-j** are presented as mean values ± SD. Statistical analyses are two-tailed unpaired student t-test. **a**, p=0.0027, **d**, p=0.0439 (30min), p=0.0414 (60min), **e**, p=0.0005 (15min), p=0.0010 (30min), p=0.0002 (60min), p=0.0147 (120min), **f**, p=0.0371 (15min), p=0.0078 (30min), p=0.0055 (60min), p=0.0127 (120min). **j**, p=0.0006. * p<0.05, ** p<0.01, ***p<0.001 and NS; not significant. Source data are provided as a Source Data file.

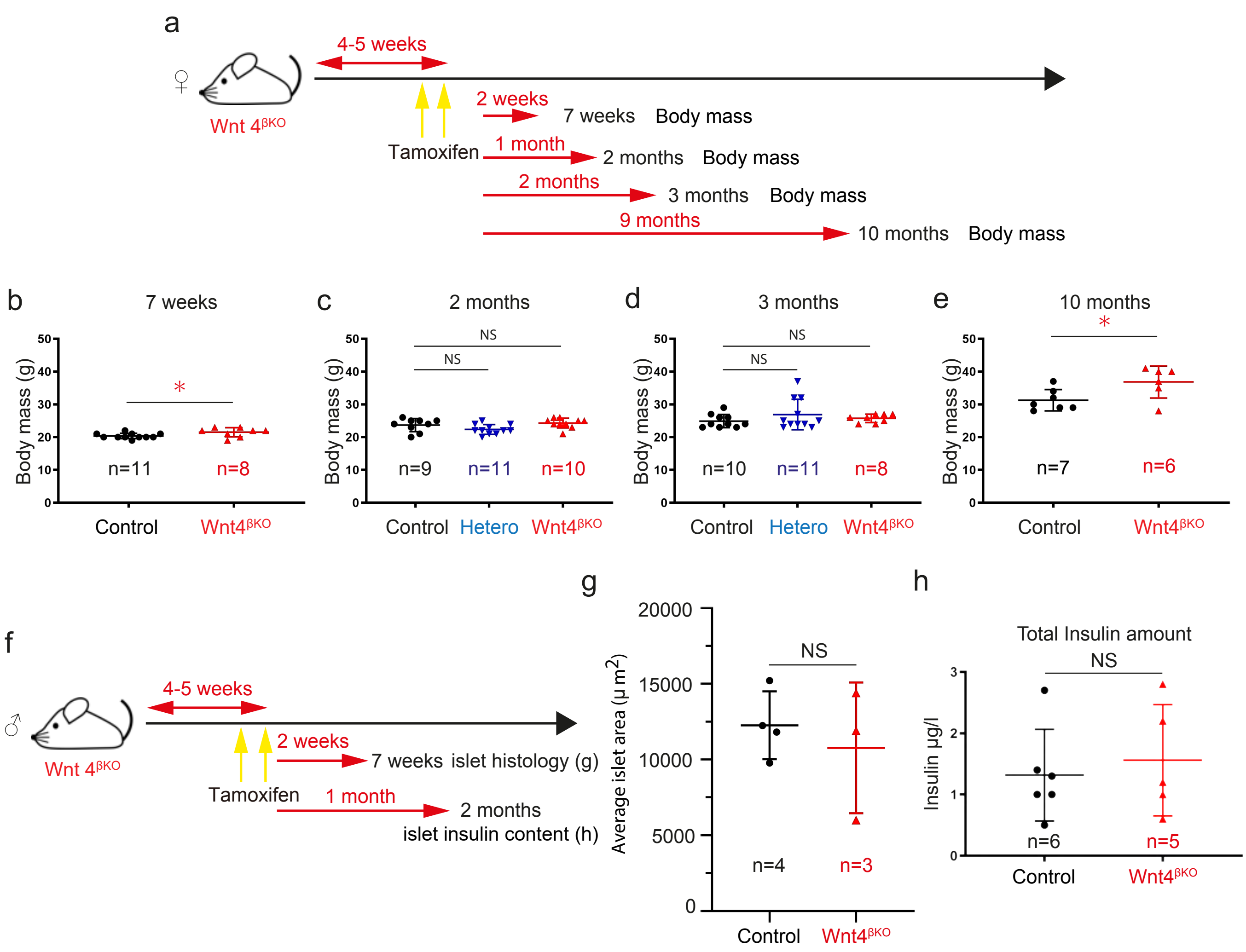

Supplementary Fig. 5: Body mass in *Wnt4$^{\beta KO}$* females and islet area and insulin content in males. **a**, Illustration of experimental design: after the tamoxifen-induced inactivation of *Wnt4* in β-cells in females, the body mass was measured in the mice submitted to IPGTT reported in Supplementary Fig. 4 at 7 weeks (**b**), 2 months (**c**), 3 months (**d**) and 10 months (**e**). **f**, Experimental design relative to the islet histology quantifications presented in (**g**) and the insulin content measurements shown in (**h**). **g**, Average islet area in analysis of insulin+ and glucagon+ cell ratio and insulin+ and glucagon+ average cell area (Fig. 4**d**,**e**). **h**, Total insulin amount of islet assessed by ELISA, relative to the insulin secretion shown in Fig. 4**g**. Data in graph of **b-e, g-h** are presented as mean values ± SD. Statistical analyses are two-tailed unpaired student t-test. **b**, p=0.0399, **e**, p=0.0322. *p<0.05 and NS; not significant. Source data are provided as a Source Data file.

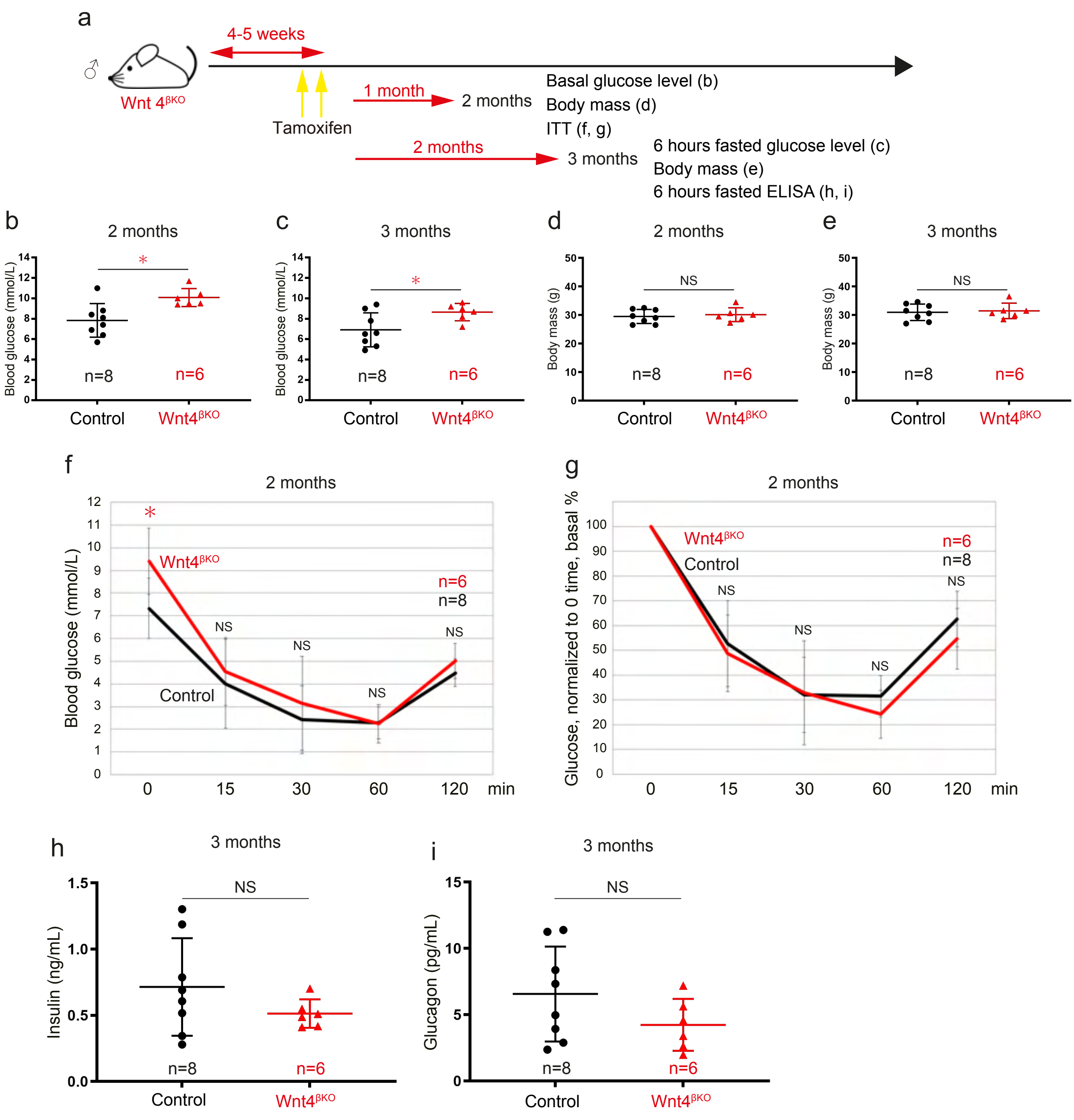

Supplementary Fig. 6: Insulin tolerance test and fasted insulin and glucagon amount in *Wnt4$^{\beta KO}$* mice. **a**, Experimental design for basal glucose (**b**), body mass (**d**) and insulin tolerance test (ITT) (**f,g**) at 2 months and 6 hours fasted glucose (**c**), body mass (**e**), ELISA measurement of insulin (**h**) and glucagon (**i**) at 3 months in tamoxifen-induced *Wnt4* inactivation in β-cells in male mice. **b**, Basal blood glucose level at 2 months. **c**, 6 hours fasted blood glucose level at 3 months. **d,e**, Body mass of *Wnt4$^{\beta KO}$* male mice at 2 months (**d**) and 3 months (**e**). **f,g,** Result of insulin tolerance test (ITT) of *Wnt4$^{\beta KO}$* male mice at 2 months (**f**) and glucose level was normalized to 0 time (**g**). **h**,**i**, ELISA for insulin (**h**) and glucagon (**i**) in 6 hours fasted blood samples from 3 months *Wnt4$^{\beta KO}$* male mice. Data in graph of **b-i** are presented as mean values ± SD. Statistical analyses are two-tailed unpaired student t-test. **b**, p=0.0108, **c**, p=0.0389, **f**, p=0.0169 (0 time), *p<0.05 and NS; not significant. Source data are provided as a Source Data file.

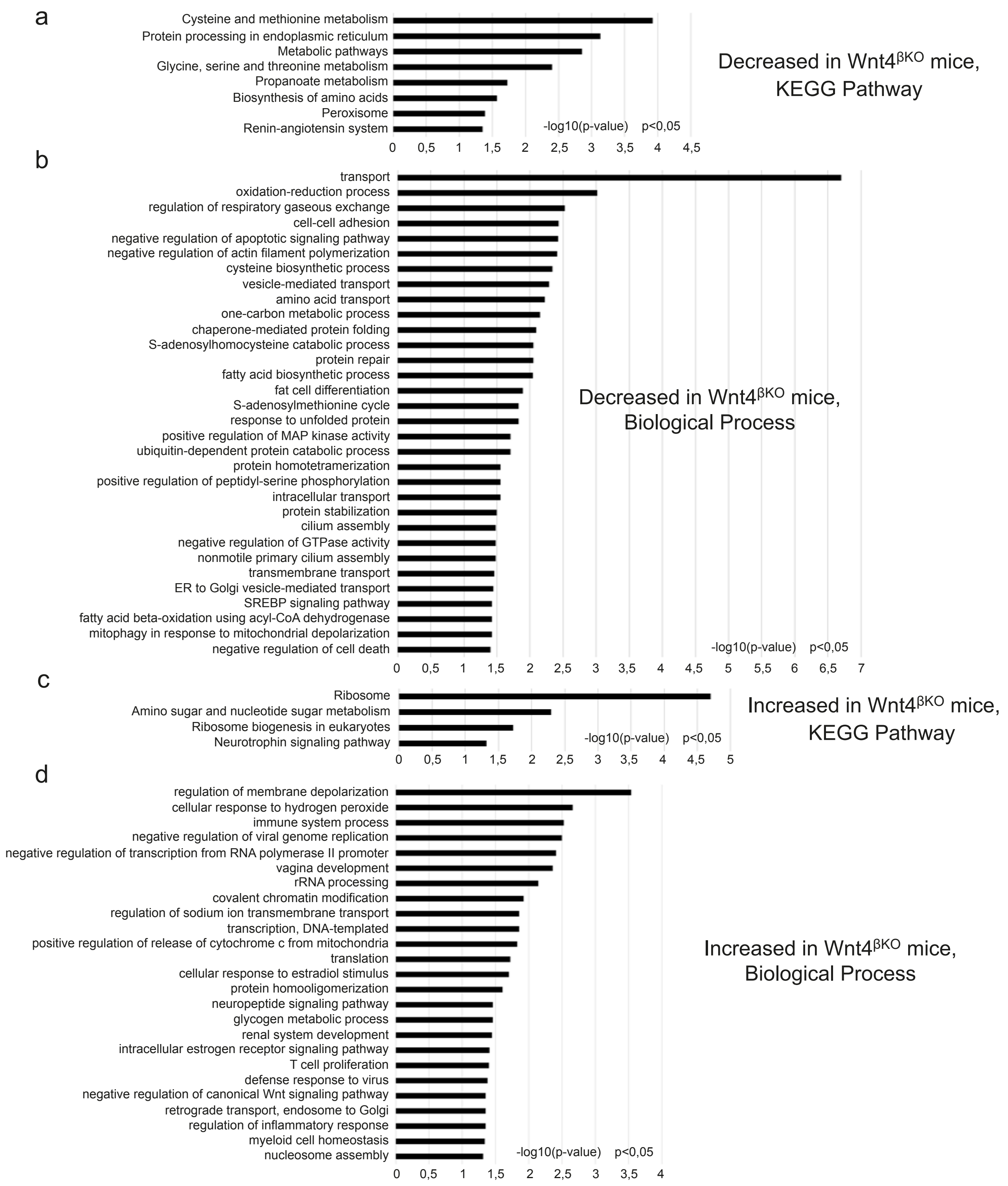

Supplementary Fig. 7: Gene ontology analysis of 7-weeks *Wnt4$^{\beta KO}$* islets.
a-d, Gene ontology analysis results of 7-weeks *Wnt4$^{\beta KO}$* islets based on DAVID Bioinformatics Resources 6.8. Results with the modified Fisher exact p-value (EASE score) < 0.05 were considered to be significantly enriched. Selected significant enriched terms of gene ontology. Enriched terms of KEGG Pathway (**a**) and Biological Process (**b**) for down-regulated genes in *Wnt4$^{\beta KO}$* islets. Enriched terms of KEGG Pathway (**c**) and Biological Process (**d**) for up-regulated genes in *Wnt4$^{\beta KO}$* islets. Source data are provided as a Source Data file.

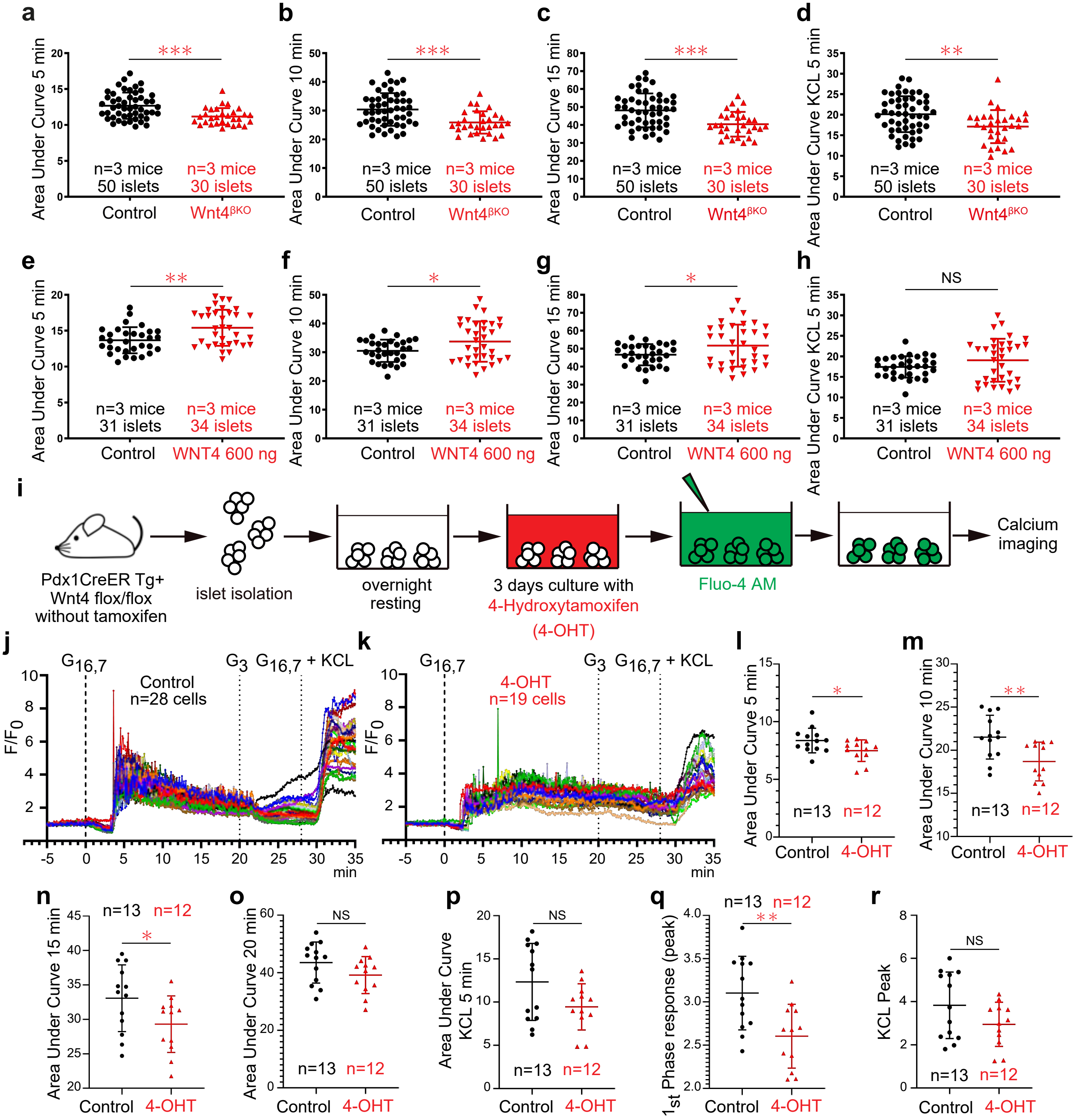

Supplementary Fig. 8: WNT4 regulates influx of calcium in mouse islets together with glucose. **a-d**, Area Under Curve (AUC) of calcium fluorescence traces in 2 months *Wnt4^{βKO}* islets (Related to Fig. 6**b**) at 5 min in high glucose (**a**), 10 min in high glucose (**b**), 15 min in high glucose (**c**) and 5 min of KCl (**d**). **e-h**, AUC of calcium fluorescence trace in the absence or presence of WNT4 (Related to Fig. 6**e**) at 5 min in high glucose (**e**), 10 min in high glucose (**f**), 15 min in high glucose (**g**) and 5 min of KCl (**h**). **i-r,** Imaging calcium in islets after *in vitro Wnt4* inactivation. **i,** Experimental design for *in vitro* calcium imaging. Single cell imaging influx of calcium in control (**j**) and *Wnt4* inactivation (4-OHT, **k**). **l-r**, Islets imaging influx of calcium in control (black) and *Wnt4* inactivation (4-OHT, Red). AUC of calcium trace after *in vitro* inactivation of *Wnt4* in islets after 5 min in high glucose (**l**), 10 min in high glucose (**m**), 15 min in high glucose **(n**), 20 min in high glucose (**o**), and 5 min in KCl (**p**), 1st phase response peak (**q**) and peak in KCl (**r**). Data in graph of **a-h**, **l-r** are presented as mean values ± SD. Statistical analyses are two-tailed unpaired student t-test. **a**, p=0.00002, **b**, p=0.00006, **c**, p=0.0002, **d**, p=0.0029, **e**, p=0.0024, **f**, p=0.0241, **g**, p=0.0302, **l,** p=0.0392, **m**, p=0.0073, **n**, p=0.0480, **q**, p=0.0048. *p<0.05, **p<0.01, ***p<0.001 and NS; not significant. Source data are provided as a Source Data file.

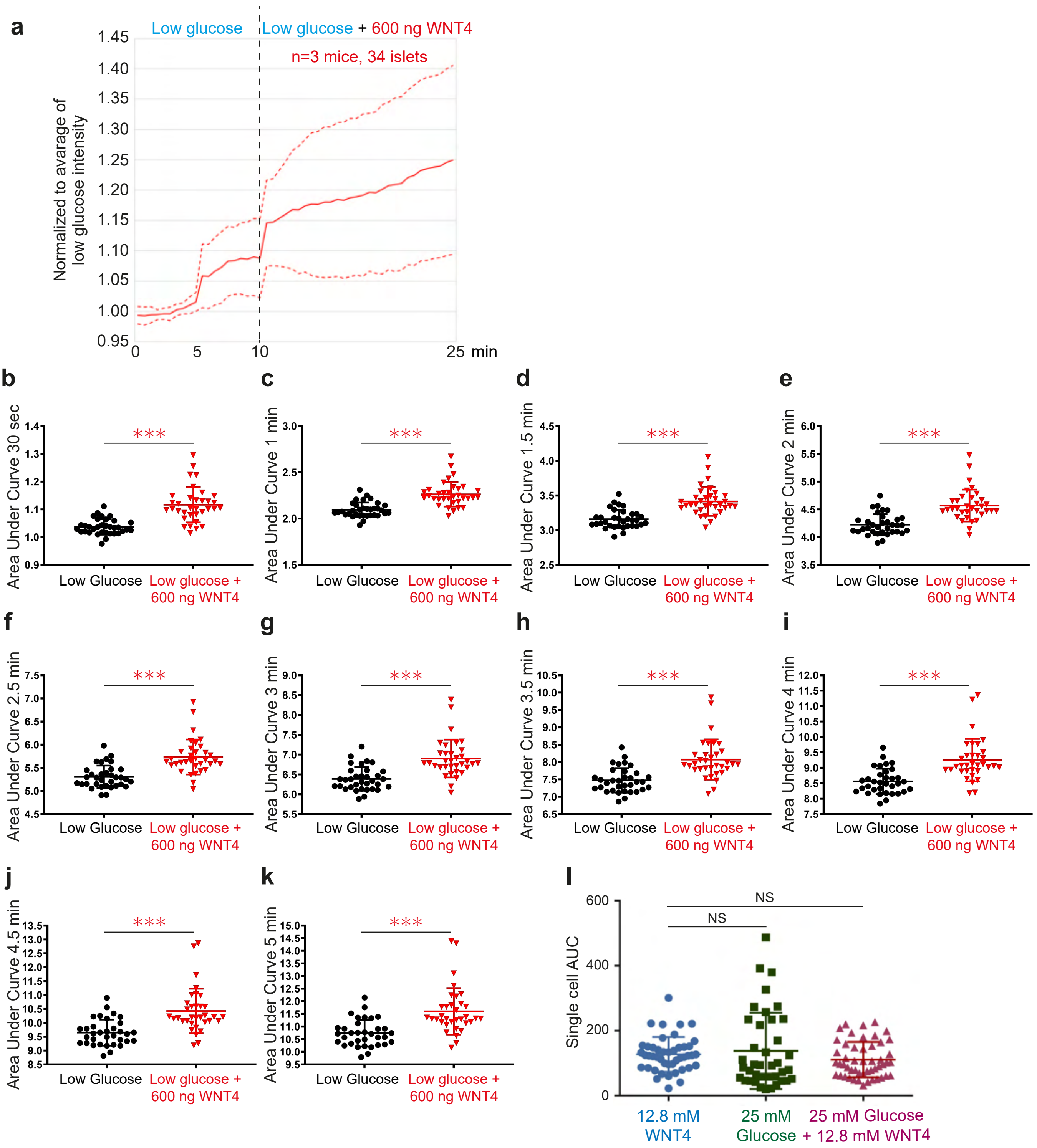

Supplementary Fig. 9: WNT4 alone positively regulate influx of calcium in mouse islets.
a, Calcium signaling in the presence of 600 ng WNT4 in low glucose. Red broken lines show standard deviations. **b-k**, AUC of calcium fluorescence trace in 600 ng WNT4 in low glucose (**a**) at 30 sec (**b**), at 1 min (**c**), at 1.5 min (**d**), at 2 min (**e**), at 2.5 min (**f**), at 3 min (**g**), at 3.5 min (**h**), at 4 min (**i**), at 4.5 min (**j**) and 5 min (**k**). **a-k**, 34 islets from 3 mice were analyzed. Data in graph of **a-k** are presented as mean values ± SD. Statistical analyses are two-tailed paired student t-test. **b**, p=9.54e-11, **c**, p=5.92e-10, **d**, p=1.11e-09, **e**, p=1.9e-09, **f**, p=4.07e-09, **g**, p=1.11e-08, **h**, p=2.65e-08, **i**, p=5.4e-08, **j**, p=1.03e-07, **k**, p=1.68e-07. ***p<0.001. **l**, AUC of single cell imaging influx of calcium in Zebrafish in Fig. 6**f-i**. 45 cells, n=4 larvae (12.8 mM WNT4), 39 cells, n=4 larvae (25 mM Glucose), 50 cells, n=5 larvae (25 mM Glucose + 12.8 mM WNT4). Data are mean ± s.d (1-way –ANOVA with Tukey's multiple comparison correction, NS, not significant). Source data are provided as a Source Data file.

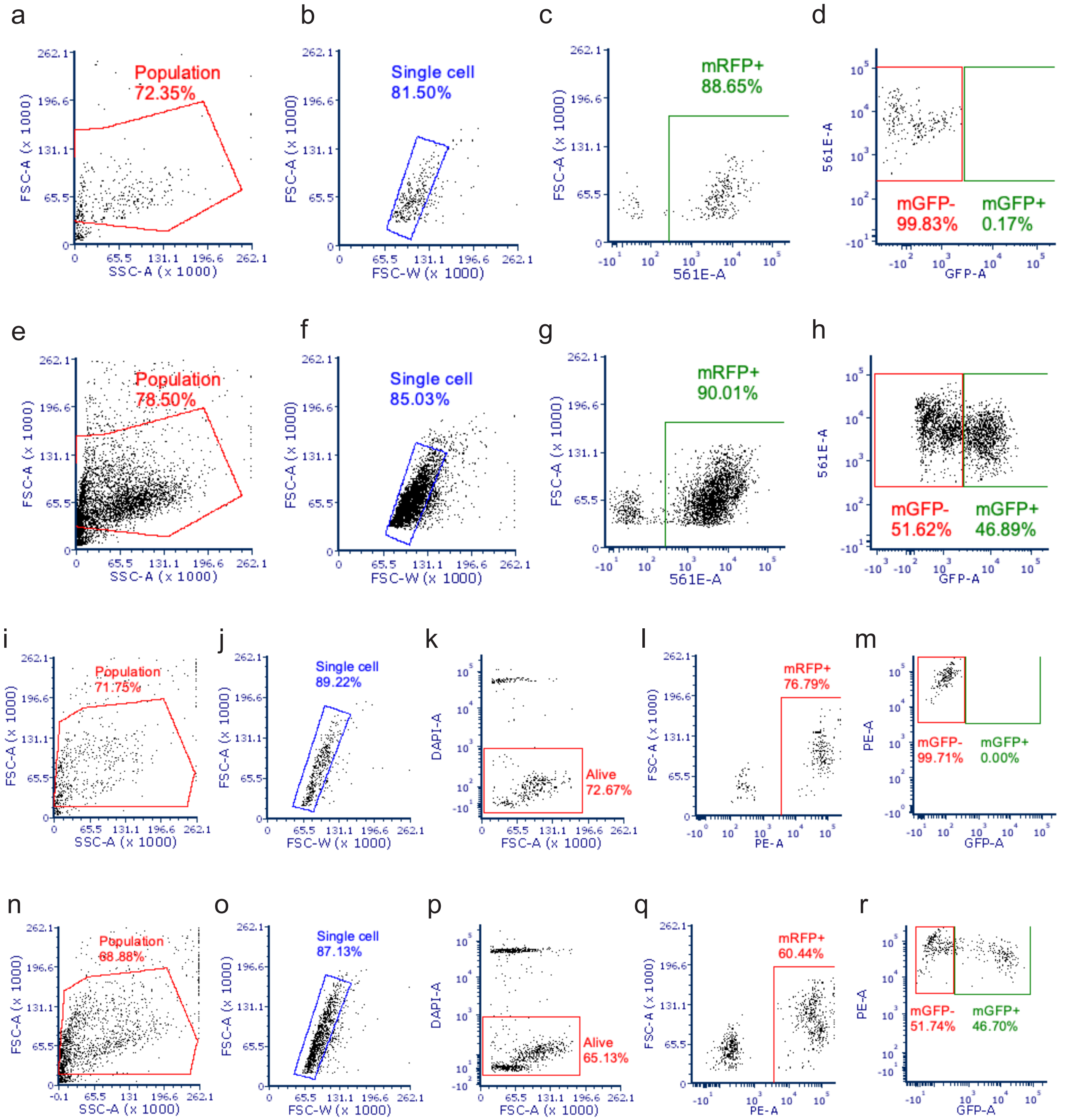

Supplementary Fig. 10: Gating Strategy of Flow Cytometry.
a-h, Gating strategy of mGFP+ islet cells and mGFP- islet cells in cell proliferation analysis (Fig. 2**f-i**). **a-d**, Control islets. **e-h**, Mutant islets. Cell population (**a,e**), single cells (**b,f**), mRFP+ islet cells (**c,g**) and mGFP+ islet cells and mGFP- islet cells (**d,h**). **i-r**, Gating strategy of mGFP+ islet cells and mGFP- islet cells in mitochondrial mass analysis (Fig. 2**j-n**). **i-m**, Control islets. **n-r**, Mutant islets. Cell population (**i,n**), single cells (**j,o**), live cells (**k,p**), mRFP+ islet cells (**l,q**) and mGFP+ islet cells and mGFP- islet cells (**m,r**).
