# Supplementary material for: Wnt4 is heterogeneously activated in maturing β-cells to control calcium signaling, metabolism and function
Source: Nat Commun. 2022 Oct 21;13:6255. doi: 10.1038/s41467-022-33841-5 (PMC9587236; doi:10.1038/s41467-022-33841-5)
Supplement: Supplementary file 2 — Description of Additional Supplementary Files [file 41467_2022_33841_MOESM2_ESM.pdf]

Description of Additional Supplementary Files

File Name: Supplementary Data 1
Description: Transcriptome analysis of the Wnt4hi and Wnt4low populations at P1
Statistical analysis is derived using empirical Bayes statistical analysis. p-value < 0.05.

File Name: Supplementary Data 2
Description: Transcriptome analysis of 7-week Wnt4βKO and WT islets
Statistical analysis is derived using empirical Bayes statistical analysis. p-value < 0.05.
