# Supplementary material for: Wnt4 is heterogeneously activated in maturing β-cells to control calcium signaling, metabolism and function
Source: Nat Commun. 2022 Oct 21;13:6255. doi: 10.1038/s41467-022-33841-5 (PMC9587236; doi:10.1038/s41467-022-33841-5)
Supplement: Supplementary file 5 — Reporting Summary [file 41467_2022_33841_MOESM5_ESM.pdf]

nature portfolio

Corresponding author(s): Anne Grapin-Botton

Last updated by author(s): October 1, 2022

# Reporting Summary

Nature Portfolio wishes to improve the reproducibility of the work that we publish. This form provides structure for consistency and transparency in reporting. For further information on Nature Portfolio policies, see our Editorial Policies and the Editorial Policy Checklist.

## Statistics

For all statistical analyses, confirm that the following items are present in the figure legend, table legend, main text, or Methods section.

| n/a | Confirmed | |
|---|---|---|
| ☐ | ☒ | The exact sample size ($n$) for each experimental group/condition, given as a discrete number and unit of measurement |
| ☐ | ☒ | A statement on whether measurements were taken from distinct samples or whether the same sample was measured repeatedly |
| ☐ | ☒ | The statistical test(s) used AND whether they are one- or two-sided<br>*Only common tests should be described solely by name; describe more complex techniques in the Methods section.* |
| ☒ | ☐ | A description of all covariates tested |
| ☐ | ☒ | A description of any assumptions or corrections, such as tests of normality and adjustment for multiple comparisons |
| ☐ | ☒ | A full description of the statistical parameters including central tendency (e.g. means) or other basic estimates (e.g. regression coefficient) AND variation (e.g. standard deviation) or associated estimates of uncertainty (e.g. confidence intervals) |
| ☐ | ☒ | For null hypothesis testing, the test statistic (e.g. $F$, $t$, $r$) with confidence intervals, effect sizes, degrees of freedom and $P$ value noted<br>*Give P values as exact values whenever suitable.* |
| ☒ | ☐ | For Bayesian analysis, information on the choice of priors and Markov chain Monte Carlo settings |
| ☒ | ☐ | For hierarchical and complex designs, identification of the appropriate level for tests and full reporting of outcomes |
| ☒ | ☐ | Estimates of effect sizes (e.g. Cohen's $d$, Pearson's $r$), indicating how they were calculated |

*Our web collection on statistics for biologists contains articles on many of the points above.*

## Software and code

Policy information about availability of computer code

| | |
|---|---|
| Data collection | Mouse database: Pyrat v4.5.1-383<br>Microsoft Excel v16.65<br>Zeiss ZEN Microscopy Software v14.0.25.201<br>Leica Application Suite X (LAS X) v3.5.5.19976<br>BD FACSDiva™ software v8.0.1 |
| Data analysis | Fiji/Image J v2.1.0/1.53c<br>Express 6 Flow Research Edition software v6.01 (De Novo Software).<br>R-BioConductor package limma v3.28.14<br>David online tool (https://david.ncifcrf.gov)<br>MassLynx software (Waters)<br>GraphPad Prism 6 v9.4.0<br>Microsoft Excel software v16.65 |

For manuscripts utilizing custom algorithms or software that are central to the research but not yet described in published literature, software must be made available to editors and reviewers. We strongly encourage code deposition in a community repository (e.g. GitHub). See the Nature Portfolio guidelines for submitting code & software for further information.

# Data

Policy information about availability of data

All manuscripts must include a data availability statement. This statement should provide the following information, where applicable:

- Accession codes, unique identifiers, or web links for publicly available datasets
- A description of any restrictions on data availability
- For clinical datasets or third party data, please ensure that the statement adheres to our policy

Transcriptome data are available at Gene Expression Omnibus (Geo) under accession numbers: GSE210237 (P1 GFP+ vs GFP- samples) and GSE210267 (conditional inducible knock-out of Wnt4 in β-cells). Metabolic prolifiling data are available in MetaboLights under number MTBLS6012.

# Human research participants

Policy information about studies involving human research participants and Sex and Gender in Research.

| | |
|---|---|
| Reporting on sex and gender | not applicable |
| Population characteristics | not applicable |
| Recruitment | not applicable |
| Ethics oversight | not applicable |

Note that full information on the approval of the study protocol must also be provided in the manuscript.

# Field-specific reporting

Please select the one below that is the best fit for your research. If you are not sure, read the appropriate sections before making your selection.

☒ Life sciences ☐ Behavioural & social sciences ☐ Ecological, evolutionary & environmental sciences

For a reference copy of the document with all sections, see nature.com/documents/nr-reporting-summary-flat.pdf

# Life sciences study design

All studies must disclose on these points even when the disclosure is negative.

| | |
|---|---|
| Sample size | Sample size calculations could not be performed at study onset as none of the observed effects had been reported before. It was set according to common practice in the field. A minimum was set at three but most commonly 4 or 5 samples. For the glucose tolerance tests, previous work has shown that higher numbers are needed to reach statistical significance due to variability between individuals. We used a minimum number of 6 individuals. Sample numbers are indicated in the figure legends as N independent experiments containing n samples and can be seen from individual point representations on the graphs. |
| Data exclusions | For the microarray experiments comparing the Wnt4 KO in beta cells and the control, we initially had 4 samples of each category. We excluded one control sample as it did not pass the quality tests. |
| Replication | All data were replicated in independent experiments (indicated as N in the figure legends) and no experiment was excluded. |
| Randomization | All treatment conditions during in vitro experiments were allocated randomly.  For immunohistochemistry, the field of view was randomly selected for analysis. |
| Blinding | Blinding was not possible as experimental conditions were often evident from the image or FACS data. Quantifications were performed using computational pipeline applied equally to all conditions and replicates. Thresholds were applied similarly to all samples. Quantitative measurements should limit investigator bias. |

# Reporting for specific materials, systems and methods

We require information from authors about some types of materials, experimental systems and methods used in many studies. Here, indicate whether each material, system or method listed is relevant to your study. If you are not sure if a list item applies to your research, read the appropriate section before selecting a response.

## Materials & experimental systems

| n/a | Involved in the study |
|---|---|
| ☐ | ☒ Antibodies |
| ☒ | ☐ Eukaryotic cell lines |
| ☒ | ☐ Palaeontology and archaeology |
| ☐ | ☒ Animals and other organisms |
| ☒ | ☐ Clinical data |
| ☒ | ☐ Dual use research of concern |

## Methods

| n/a | Involved in the study |
|---|---|
| ☒ | ☐ ChIP-seq |
| ☐ | ☒ Flow cytometry |
| ☒ | ☐ MRI-based neuroimaging |

# Antibodies

| | |
|---|---|
| Antibodies used | These antibodies were used in this study for immunohistochemistry; Primary antibodies were goat anti-WNT4 (R&D system, AF475, 1:100)(Fig. 1g), rabbit anti-WNT4 (Bioss Antibodies, BS-6134R, 1:100)(Extended Data Fig. 3), guinea pig anti-Insulin (DAKO, A0564, 1:100), mouse anti-Glucagon (Sigma, G2654, 1:800), chicken anti-GFP (Abcam, ab13970, 1:1000), rabbit anti-Ki67 (Abcam, ab16667, 1:100), mouse anti-active beta-catenin (Millipore, 05-665, 1:100), rabbit anti-pMLC (Cell Signaling Technology, 3674, 1:100). Secondary antibodies were donkey anti-mouse Alexa Fluor568 (Thermo Fisher, A10037, 1:1000), donkey anti-mouse Alexa Fluor647 (Jackson ImmunoResearch Europe Ltd, 715-605-150, 1:800), donkey anti-chicken Alexa Fluor488 (Jackson ImmunoResearch Europe Ltd, 703-545-155, 1:800), goat anti-chicken Alexa Fluor488 (Thermo Fisher, A-11039, 1:1000), donkey anti-goat Alexa Fluor488 (Abcam, ab150129, 1:1000), donkey anti-guinea pig Texas red (Abcam, ab6906, 1:300), donkey anti-guinea pig Biotin (Jackson ImmunoResearch Europe Ltd, 706-065-1480, 1:400), donkey anti-rabbit Biotin (Jackson ImmunoResearch Europe Ltd, 711-065-152, 1:200), Streptavidin Alexa Fluor647 (Jackson ImmunoResearch Europe Ltd, 016-600-0840, 1:1000). Nuclei were stained with DAPI (Sigma, D9542-1MG, 1:10000). |
| Validation | All antibodies have been validated by manufacturers at least by Western blot. The secondary antibodies have been reliable with multiple primary antibodies in our hands. They have also been reported in numerous other studies in mouse and we have ascertained their tissue distribution and subcellular localization. A test with no primary antibody was always included in the initial antibody validations by immunohistochemistry. Rabbit anti-WNT4 (Bioss Antibodies, BS-6134R, 1:100) was further validated by negative staining in the Wnt4 KO beta cells. |

# Animals and other research organisms

Policy information about studies involving animals; ARRIVE guidelines recommended for reporting animal research, and Sex and Gender in Research

| | |
|---|---|
| Laboratory animals | Mouse lines:<br>To generate mouse samples, breeders younger than 2 years old were used.<br>: Tg(Ifp1-cre/Esr1)35.10Dam 47 (Pdx1CreER), Wnt4tm1Svo/tm1Svo 48 (Wnt4 fl/fl), Gt(ROSA)26Sortm4(ACTB−tdTomato,−EGFP)Lu49 (mTmG), Gt(ROSA)26Sor (Rosa26LacZ), Wnt4tm2(EGFP/cre)Svo32 (Wnt4eGFPCre), Pdx1-CreER;Wnt4 fl/fl mice (Wnt4βKO), Pdx1CreER;Wnt4 fl/fl;Rosa26LacZ (Wnt4βKO;LacZ), Pdx1CreER;Wnt4 fl/fl;mTmG (Wnt4βKO;mTmG). Specific age of collection is indicated in the manuscript for each experiment.<br><br>Zebrafish lines:<br>To generate zebrafish samples, less than 1 year old zebrafish (3-9 months) are used to mate.<br>All the experiments were conducted in larvae less than 5 days post fertilization.<br>Tg(ins:gCaMP6s;cryaa:mCherry)53 , Tg(ins:cdt1-mCherry;cryaa:GFP)54 |
| Wild animals | None |
| Reporting on sex | The data were collected on males and females. Specific information relative to gender is indicated for each experiment. |
| Field-collected samples | None |
| Ethics oversight | Mouse experiments:<br>All animal experiments were performed under license 2014-15-2934-01008 and 2019-15-0201-01613 from the Danish Veterinary Office and the license of Tierversuchsvorhabens (TVV 9/2020, “Wnt4 im Pankreas”) from the Governmental IACUC (“Landesdirektion Sachsen”). And all experiments were approved by the Danish Animal Experiments Inspectorate (Dyreforsøgstilsynet), the Governmental IACUC (“Landesdirektion Sachsen”) and the Max Planck institute Animal Welfare Officer.<br>Zebrafish experiments:<br>All experiments were carried out in compliance with European Union and German laws (Tierschutzgesetz) and with the approval of the TU Dresden and the Landesdirektion Sachsen Ethics Committees (approval no: TVV 45/2018). In this study, all live imaging in vivo, compound and glucose injections, as well as experimental procedures were performed with zebrafish larvae that did not exceed the 5-dpf stage, as stated in the animal protection law (TierSchVersV §14). According to the EU directive 2010/63/EU, the use of these earlier zebrafish stages reduces the number of experimental animals, according to the principles of the 3Rs. |

Note that full information on the approval of the study protocol must also be provided in the manuscript.

## Flow Cytometry

### Plots

Confirm that:

☒ The axis labels state the marker and fluorochrome used (e.g. CD4-FITC).

☒ The axis scales are clearly visible. Include numbers along axes only for bottom left plot of group (a 'group' is an analysis of identical markers).

☒ All plots are contour plots with outliers or pseudocolor plots.

☒ A numerical value for number of cells or percentage (with statistics) is provided.

### Methodology

| | |
|---|---|
| Sample preparation | Cell cycle<br>The islets from 5 pups were pooled together into 1 sample. In total the islets from 25 pups were used, grouped in samples (n=5). Single cell dissociated P1 Wnt4eGFPCre; mTmG islets were stained with the Vybrant™ DyeCycle™ Violet Stain (Invitrogen, #V35003) for cell cycle analysis, following the manufacturer´s instructions. Cells were incubated at 37Ċ for 30 minutes at a final stain concentration of 1µM in RPMI 1640 medium supplemented with 2% FBS prior flow cytometry analysis.<br>Mitochondrial activity<br>The islets from 5 pups were pooled together into 1 sample. In total the islets from 25 pups were used, grouped in samples (n=5). Single cell dissociated P1 Wnt4eGFPCre; mTmG islets were stained with the MitoTracker Deep Red FM (Invitrogen, #M22426) to assay mitochondrial activity, following the manufacturer´s instructions. Cells were incubated at 37Ċ for 30 minutes at a final stain concentration of 25nM in RPMI 1640 medium supplemented with 2% FBS. After incubation time, cells were washed and resuspended in RPMI 1640 medium supplemented with 2% FBS containing 10 µM of DAPI to exclude dead cells, prior flow cytometry analysis.<br>Cell cycle<br>The islets from 5 pups were pooled together into 1 sample. In total the islets from 25 pups were used, grouped in samples (n=5). Single cell dissociated P1 Wnt4eGFPCre; mTmG islets were stained with the Vybrant™ DyeCycle™ Violet Stain (Invitrogen, #V35003) for cell cycle analysis, following the manufacturer´s instructions. Cells were incubated at 37Ċ for 30 minutes at a final stain concentration of 1µM in RPMI 1640 medium supplemented with 2% FBS prior flow cytometry analysis.<br>Mitochondrial activity<br>The islets from 5 pups were pooled together into 1 sample. In total the islets from 25 pups were used, grouped in samples (n=5). Single cell dissociated P1 Wnt4eGFPCre; mTmG islets were stained with the MitoTracker Deep Red FM (Invitrogen, #M22426) to assay mitochondrial activity, following the manufacturer´s instructions. Cells were incubated at 37Ċ for 30 minutes at a final stain concentration of 25nM in RPMI 1640 medium supplemented with 2% FBS. After incubation time, cells were washed and resuspended in RPMI 1640 medium supplemented with 2% FBS containing 10 µM of DAPI to exclude dead cells, prior flow cytometry analysis. |
| Instrument | FACS analyses were performed on a BD LSR Fortessa analyzer (BD Biosciences) controlled by BD FACSDiva™ software. |
| Software | BD FACSDiva™ software was used for data collection. Further analysis and quantifications were performed on the FCS Express 6 Flow Research Edition software (De Novo Software). |
| Cell population abundance | For stained samples, at least five thousand events of live singlets were recorded. Based on unstained and isotype controls, gates were set to determine the number of positive signal events. The numbers of events were used to generate proportion of positive populations. |
| Gating strategy | Gates were set based on singlets, live/dead staining, (Vybrant™ DyeCycle™ Violet Stain/DAPI) unstained control, and isotype control to set positive gates of live single cells. |

☐X Tick this box to confirm that a figure exemplifying the gating strategy is provided in the Supplementary Information.
